# Supplementary material for: Optogenetic actuator – ERK biosensor circuits identify MAPK network nodes that shape ERK dynamics
Source: Mol Syst Biol. 2022 Jun 13;18(6):e10670. doi: 10.15252/msb.202110670 (PMC9189677; doi:10.15252/msb.202110670)
Supplement: Supplementary file 1 — Appendix [file MSB-18-e10670-s002.docx]

**Appendix**

**Optogenetic actuator - ERK biosensor circuits identify MAPK network nodes that shape ERK dynamics**

Coralie Dessauges^1^, Jan Mikelson^2^, Maciej Dobrzyński^1^, Marc-Antoine Jacques^1^, Agne Frismantiene^1^, Paolo Armando Gagliardi^1^, Mustafa Khammash^2^, Olivier Pertz^1,*^

^1^Institute of Cell Biology, University of Bern, Baltzerstrasse 4, 3012 Bern, Switzerland

^2^Department of Biosystems Science and Engineering, ETH Zurich, Mattenstrasse 26, 4058 Basel, Switzerland

* Corresponding author: olivier.pertz@unibe.ch

Table of Contents

[Appendix Figures 2](#_Toc102973660)

[Appendix Tables 4](#_Toc102973661)

## Appendix Figures

**Appendix Figure S1: An optogenetic actuator-biosensor genetic circuit to study input-dependent ERK dynamics. (A)** Average ERK responses of cells stimulated with a transient optoFGFR input (D = 18 mJ/cm^2^) under increasing concentrations of FGFR (SU5402), B/CRAF (RAF709), MEK (U0126) and ERK (SCH772984) inhibitors. ERK responses were normalized to the 0 μM condition for each drug (N_min_ = 40 cells per condition). **(B-C)** Quantification of optoFGFR dimerization in response to a transient 470 nm light input using a mScarlet tagged version of optoFGFR. **(B)** Cells expressing optoFGFR-mScarlet were imaged every 20 seconds with a 100x TIRF objective. Segmentation of optoFGFR dimers/oligomers was performed with the pixel classification module of Ilastik to specifically segment optoFGFR dimer-/oligomerization events (blue arrows) and exclude the endocytic vesicles (red arrows). Scale bar: 5 μm (cell) and 2 μm (FOVs). **(C)** OptoFGFR dimer-/oligomerization dynamics was quantified by computing the mean of pixel intensities from the binarized mask obtained with Ilastik. Single-cell trajectories were normalized to the baseline before stimulation (N = 12). **(D)** Investigation of the “dip” (green rectangle) observed in ERK trajectories in response to a transient optoFGFR input. The dip was not suppressed by ERK inhibition (10 μM SCH772984) but was abolished under Calcineurin inhibition (100 μg/ml Cyclosporin A (CsA)). **(E)** Schematized representation of MAPK and Ca^2+^ signaling effect on the ERK-KTR reporter dynamics. **(F)** Validation of Ca^2+^ signaling effect on ERK-KTR by chemically increasing the intracellular Ca^2+^ concentration with 1 μM Ionomycin.

## Appendix Tables

| **Species** | **Notation** | **Initial Value** |
| --- | --- | --- |
| RAS | $RAS$ | 1 |
| Phosphorylated RAS | ${RAS}^{\text{*}}$ | 0 |
| RAF | $RAF$ | 1 |
| Phosphorylated RAF | ${RAF}^{\text{*}}$ | 0 |
| MEK | $MEK$ | 1 |
| Phosphorylated MEK | ${MEK}^{\text{*}}$ | 0 |
| ERK | $ERK$ | 1 |
| Phosphorylated ERK | ${ERK}^{\text{*}}$ | 0 |
| EGF receptor | $EGFR$ | 1 |
| Active EGF receptor | ${EGFR}^{\text{*}}$ | 0 |
| Endocytosed EGF receptor | ${EGFR}_{endo}$ | 0 |
| Negative feedback species | $NFB$ | 1 |
| Active negative feedback species | ${NFB}^{\text{*}}$ | 0 |
| nuclear KTR | $KTR$ | $1-{ktr}_{init}$ |
| cytosolic KTR | ${KTR}^{\text{*}}$ | ${ktr}_{init}$ |
| EGF input | $EGF$ | Input variable |
| Light input | $light$ | Input variable |

**Appendix Table S1: Model species, notations, and initial values**

| **Nbr** | **Model Equations** |
| --- | --- |
| 1 | $EGFR=-r_{1,2}EGFEGFR+r_{2,1}{EGFR}^{\text{*}}+r_{3,1}{EGFR}_{endo}$ |
| 2 | ${EGFR}^{\text{*}}=r_{1,2}EGFEGFR-\left( r_{2,1}+r_{2,3} \right){EGFR}^{\text{*}}$ |
| 3 | ${EGFR}_{endo}=r_{2,3}{EGFR}^{\text{*}}-r_{3,1}{EGFR}_{endo}$ |
| 4 | $RAS=-\left( k_{1,2}{EGFR}^{\text{*}}+light \right)\frac{RAS}{K_{1,2}+RAS}+k_{2,1}\frac{{RAS}^{\text{*}}}{K_{2,1}+{RAS}^{\text{*}}}$ |
| 5 | ${RAS}^{\text{*}}=\left( k_{1,2}{EGFR}^{\text{*}}+light \right)\frac{RAS}{K_{1,2}+RAS}-k_{2,1}\frac{{RAS}^{\text{*}}}{K_{2,1}+{RAS}^{\text{*}}}$ |
| 6 | $RAF=-k_{3,4}{Ras}^{\text{*}}\frac{Raf}{K_{3,4}+Raf}+\left( k_{nfb}{NFB}^{\text{*}}+k_{4,3} \right)\frac{{Raf}^{\text{*}}}{K_{4,3}+{Raf}^{\text{*}}}$ |
| 7 | ${RAF}^{\text{*}}=k_{3,4}{Ras}^{\text{*}}\frac{Raf}{K_{3,4}+Raf}-\left( k_{nfb}{NFB}^{\text{*}}+k_{4,3} \right)\frac{{Raf}^{\text{*}}}{K_{4,3}+{Raf}^{\text{*}}}$ |
| 8 | $MEK=-k_{5,6}\cdot{RAF}^{\text{*}}\frac{MEK}{K_{5,6}+MEK}+k_{6,5}\frac{{MEK}^{\text{*}}}{K_{6,5}+{MEK}^{\text{*}}}$ |
| 9 | ${MEK}^{\text{*}}=k_{5,6}\cdot{RAF}^{\text{*}}\frac{MEK}{K_{5,6}+MEK}-k_{6,5}\frac{{MEK}^{\text{*}}}{K_{6,5}+{MEK}^{\text{*}}}$ |
| 10 | $ERK=-k_{7,8}\cdot{MEK}^{\text{*}}\frac{ERK}{K_{7,8}+ERK}+k_{8,7}\frac{{ERK}^{\text{*}}}{K_{8,7}+{ERK}^{\text{*}}}$ |
| 11 | ${ERK}^{\text{*}}=k_{7,8}\cdot{MEK}^{\text{*}}\frac{ERK}{K_{7,8}+ERK}-k_{8,7}\frac{{ERK}^{\text{*}}}{K_{8,7}+{ERK}^{\text{*}}}$ |
| 12 | $NFB=-f_{1,2}\cdot{ERK}^{\text{*}}\frac{NFB}{F_{1,2}+NFB}+f_{2,1}\frac{{NFB}^{\text{*}}}{F_{2,1}+{NFB}^{\text{*}}}$ |
| 13 | ${NFB}^{\text{*}}=f_{1,2}\cdot{ERK}^{\text{*}}\frac{NFB}{F_{1,2}+NFB}-f_{2,1}\frac{{NFB}^{\text{*}}}{F_{2,1}+{NFB}^{\text{*}}}$ |
| 14 | $KTR=-\left( k_{9,10}\cdot{ERK}^{\text{*}}\frac{KTR}{K_{9,10}+KTR}+s_{1,2}KTR \right)+s_{2,1}{KTR}^{\text{*}}$ |
| 15 | ${KTR}^{\text{*}}=\left( k_{9,10}\cdot{ERK}^{\text{*}}\frac{KTR}{K_{9,10}+KTR}+s_{1,2}KTR \right)-s_{2,1}{KTR}^{\text{*}}$ |

**Appendix Table S2: Model equations**

| **Nbr** | **Parameter Name** | **Description** |
| --- | --- | --- |
| 1 | $r_{1,2}$ | EGF dependent EGFR activation rate |
| 2 | $r_{2,3}$ | EGFR endocytosis rate |
| 3 | $r_{3,1}$ | EGFR recycling rate |
| 4 | $r_{2,1}$ | EGFR deactivation rate |
| 5 | $k_{1,2}$ | EGFR dependent RAS phosphorylation rate |
| 6 | $K_{1,2}$ | Michaelis constant RAS phosphorylation |
| 7 | $k_{2,1}$ | RAS dephosphorylation rate |
| 8 | $K_{2,1}$ | Michaelis constant RAS dephosphorylation |
| 9 | $k_{3,4}$ | RAF phosphorylation rate |
| 10 | $K_{3,4}$ | Michaelis constant RAF phosphorylation |
| 11 | $k_{nfb}$ | NFB effect on RAF dephosphorylation |
| 12 | $k_{4,3}$ | RAF dephosphorylation rate |
| 13 | $K_{4,3}$ | Michaelis constant RAF dephosphorylation |
| 14 | $k_{nfb}$ | NFB effect on RAF dephosphorylation |
| 15 | $k_{5,6}$ | RAF dependent MEK phosphorylation rate |
| 16 | $K_{5,6}$ | Michaelis constant MEK phosphorylation |
| 17 | $k_{6,5}$ | MEK dephosphorylation rate |
| 18 | $K_{6,5}$ | Michaelis constant MEK dephosphorylation |
| 19 | $k_{7,8}$ | MEK dependent ERK phosphorylation rate |
| 20 | $K_{7,8}$ | Michaelis constant ERK phosphorylation |
| 21 | $k_{8,7}$ | ERK dephosphorylation rate |
| 22 | $K_{8,7}$ | Michaelis constant ERK dephosphorylation |
| 23 | $k_{9,10}$ | ERK dependent KTR translocation rate |
| 24 | $K_{9,10}$ | Michaelis constant KTR translocation |
| 25 | $s_{1,2}$ | ERK independent KTR translocation rate |
| 26 | $s_{2,1}$ | nuclear KTR translocation rate |
| 27 | $f_{1,2}$ | ERK dependent NFB activation rate |
| 24 | $F_{1,2}$ | Michaelis constant NFB activation |
| 25 | $f_{2,1}$ | NFB deactivation rate |
| 26 | $F_{2,1}$ | Michaelis constant NFB deactivation |
| 27 | ${ktr}_{init}$ | Fraction of initial cytosolic KTR |
| 27 | $\sigma$ | Standard deviation of the measurement noise |

**Appendix Table S3: Model parameters**

| **siRNA name** | **Gene name** | **Mouse gene ID** | **CODEX accuracy** |  | **siRNA name** | **Gene name** | **Mouse gene ID** | **CODEX accuracy** |
| --- | --- | --- | --- | --- | --- | --- | --- | --- |
| ***FRS2*** | Frs2 | 327826 | 0.385 |  | ***RKIP*** | Pebp1 | 23980 | 0.045 |
| ***GRB2*** | Grb2 | 14784 | 0.414 |  | ***YWHAZ*** | Ywhaz | 22631 | 0.044 |
| ***GAB1*** | Gab1 | 14388 | 0.082 |  | ***YWHAG*** | Ywhag | 22628 | 0.043 |
| ***SHC1*** | Shc1 | 20416 | 0.217 |  | ***CNKSR1*** | Cnksr1 | 194231 | 0.115 |
| ***PTK2*** | Ptk2 | 14083 | 0 |  | ***KSR1*** | Ksr1 | 16706 | 0.085 |
| ***CRKL*** | Crkl | 12929 | 0.015 |  | ***PP2A*** | Ppp2ca | 19052 | 0.682 |
| ***SPRY1*** | Spry1 | 24063 | 0.113 |  | ***ARAF*** | Araf | 11836 | 0.021 |
| ***SPRY2*** | Spry2 | 24064 | 0.058 |  | ***BRAF*** | Braf | 109880 | 0.031 |
| ***SPRY3*** | Spry3 | 236576 | 0.174 |  | ***CRAF*** | Raf1 | 110157 | 0.323 |
| ***SPRY4*** | Spry4 | 24066 | 0.083 |  | ***MEK1*** | Map2k1 | 26395 | 0.056 |
| ***PTPN6*** | Ptpn6 | 15170 | 0.11 |  | ***MEK2*** | Map2k2 | 26396 | 0.054 |
| ***PTPN11*** | Ptpn11 | 19247 | 0.128 |  | ***ERK1*** | Mapk3 | 26417 | 0.032 |
| ***SHIP*** | Inpp5d | 16331 | 0.125 |  | ***ERK2*** | Mapk1 | 26413 | 0.578 |
| ***PLCG1*** | Plcg1 | 18803 | 0.589 |  | ***RSK2*** | Rps6ka3 | 110651 | 0.645 |
| ***SRC*** | Src | 20779 | 0.136 |  | ***PEA15*** | Pea15a | 18611 | 0.11 |
| ***NCK1*** | Nck1 | 17973 | 0.002 |  | ***DUSP1*** | Dusp1 | 19252 | 0.188 |
| ***NCK2*** | Nck2 | 17974 | 0.069 |  | ***DUSP2*** | Dusp2 | 13537 | 0.031 |
| ***SOS1*** | Sos1 | 20662 | 0.319 |  | ***DUSP3*** | Dusp3 | 72349 | 0.285 |
| ***SOS2*** | Sos2 | 20663 | 0.041 |  | ***DUSP4*** | Dusp4 | 319520 | 0.113 |
| ***RAPGEF1*** | Rapgef1 | 107746 | 0.385 |  | ***DUSP6*** | Dusp6 | 67603 | 0.453 |
| ***RAPGEF3*** | Rapgef3 | 223864 | 0.052 |  | ***DUSP8*** | Dusp8 | 18218 | 0.068 |
| ***RASGRP1*** | Rasgrp1 | 19419 | 0.128 |  | ***DUSP9*** | Dusp9 | 75590 | 0.164 |
| ***RASA1*** | Rasa1 | 218397 | 0 |  | ***DUSP10*** | Dusp10 | 63953 | 0.266 |
| ***NF1*** | Nf1 | 18015 | 0.112 |  | ***DUSP14*** | Dusp14 | 56405 | 0.066 |
| ***RAP1A*** | Rap1a | 109905 | 0.024 |  | ***DUSP16*** | Dusp16 | 70686 | 0.035 |
| ***RAP1B*** | Rap1b | 215449 | 0.099 |  | ***DUSP22*** | Dusp22 | 105352 | 0.012 |
| ***HRAS*** | Hras | 15461 | 0.004 |  | ***DUSP26*** | Dusp26 | 66959 | 0.155 |
| ***KRAS*** | Kras | 16653 | 0.126 |  | ***CTRL*** | - | - | 0.021 |
| ***NRAS*** | Nras | 18176 | 0.002 |  | ***ERK1+2*** | - | - | 0.711 |
| ***RRAS*** | Rras | 20130 | 0.145 |  |  |  |  |  |

**Appendix Table S4: Names of signaling nodes targeted with RNA interference and CODEX classification accuracy**

| **System** | **Inhibitor** | **EC_50_** | **Lower** | **Upper** |
| --- | --- | --- | --- | --- |
| optoFGFR | RAF709 | 7.9316 | 7.5452 | 8.3180 |
|  | U0126 | 10.3015 | 9.8784 | 10.7245 |
|  | SCH772984 | 1.1024 | 1.0456 | 1.1592 |
| optoSOS | RAF709 | 4.0766 | 3.7665 | 4.3866 |
|  | U0126 | 1.6256 | 1.4082 | 1.8430 |
|  | SCH772984 | 0.2163 | 0.1610 | 0.2717 |

**Appendix Table S5: EC_50_ with upper and lower values for the fit shown in Figure 6C**

| **System** | **Inhibitor** | **EC_50_** | **Lower** | **Upper** |
| --- | --- | --- | --- | --- |
| optoFGFR +  *CTRL* KD | RAF709 | 8.2045 | 7.5718 | 8.8373 |
|  | U0126 | 12.7216 | 11.9047 | 13.5385 |
|  | SCH772984 | 1.4052 | 1.3096 | 1.5008 |
| optoFGFR +  *RSK2* KD | RAF709 | 4.0463 | 3.6547 | 4.4380 |
|  | U0126 | 5.2965 | 4.7404 | 5.8526 |
|  | SCH772984 | 0.6369 | 0.5738 | 0.6999 |

**Appendix Table S6: EC_50_ with upper and lower values for the fit shown in Figure 6G**

| **System** | **Inhibitor** | **EC_50_** | **Lower** | **Upper** |
| --- | --- | --- | --- | --- |
| optoFGFR | RAF709 | 8.0643 | 7.2915 | 8.8371 |
|  | U0126 | 12.8672 | 11.9596 | 13.7749 |
|  | SCH772984 | 1.3799 | 1.2593 | 1.5005 |
| optoFGFR +  100 μM SL0101 | RAF709 | 3.5101 | 3.0769 | 3.9433 |
|  | U0126 | 5.7922 | 5.2616 | 6.3228 |
|  | SCH772984 | 0.4016 | 0.3363 | 0.4669 |

**Appendix Table S7: EC_50_ with upper and lower values for the fit shown in Figure EV5E**
