# Supplementary material for: Optogenetic actuator – ERK biosensor circuits identify MAPK network nodes that shape ERK dynamics
Source: Mol Syst Biol. 2022 Jun 13;18(6):e10670. doi: 10.15252/msb.202110670 (PMC9189677; doi:10.15252/msb.202110670)
Supplement: Supplementary file 2 — Expanded View Figures PDF [file MSB-18-e10670-s006.pdf]

Expanded View Figures

Figure EV1. ERK dynamics evoked by optoFGFR versus endogenous RTKs highlight different MAPK regulatory mechanisms.

A, B MaxPeak and ERKpostStim quantifications of ERK responses for each stimulation pattern shown in Fig 2C (N_{min} = 63 cells per condition). MaxPeak was quantified within a 10 min time window following the first stimulation pulse. ERKpostStim was extracted 9 min after the last pulse. Statistical analysis was done using a Wilcoxon test comparing each condition to the 20-min interval pattern (NS: non-significant, FDR *P*-value correction method).

C, D MaxPeak and FWHM quantification of ERK responses shown in Fig 3A (N_{min} = 150 cells per condition) (C) and in Fig 3B (N_{min} = 130 cells per condition) (D).

E Mathematical model topology consisting of the RAS GTPase, the MAPK three-tiered (RAF, MEK, ERK) network and the ERK-KTR reporter. EGFR and optoFGFR inputs both activate the RAS/RAF/MEK/ERK cascade, but the ERK-RAF NFB is not present. The EGFR activity is under receptor-dependent regulations.

F Simulation of ERK responses from the training dataset (Fig 3E), including the maximum a posteriori (MAP) estimate, the posterior envelope indicating the predictive density of our estimation, as well as an example trajectory.

G Predictions of the model for ERK responses evoked by 1 ng/ml EGF, 100 ng/ml EGF, and sustained high optoFGFR inputs.

H Mathematical model topology consisting of the RAS GTPase, the MAPK three-tiered (RAF, MEK, ERK) network and the ERK-KTR reporter. EGFR and optoFGFR inputs both activate the RAS/RAF/MEK/ERK cascade and the ERK-RAF NFB. The receptor-dependent regulation of EGFR was removed.

I Simulation of ERK responses from the training dataset (Fig 3E), including the maximum a posteriori (MAP) estimate, the posterior envelope indicating the predictive density of our estimation, as well as an example trajectory.

J Predictions of the model for ERK responses evoked by 1 ng/ml EGF, 100 ng/ml EGF, and sustained high optoFGFR inputs.

K ERK responses to sustained optoFGFR input (D = 18 mJ/cm^2) under dose response inhibition with the FGFR inhibitor (SU5402).

Data information: (A–D) boxes indicate the upper and lower quartiles, the central bands indicate the median, and whiskers extend to individuals up to 1.5 interquartile away from the median.

© 2022 The Authors

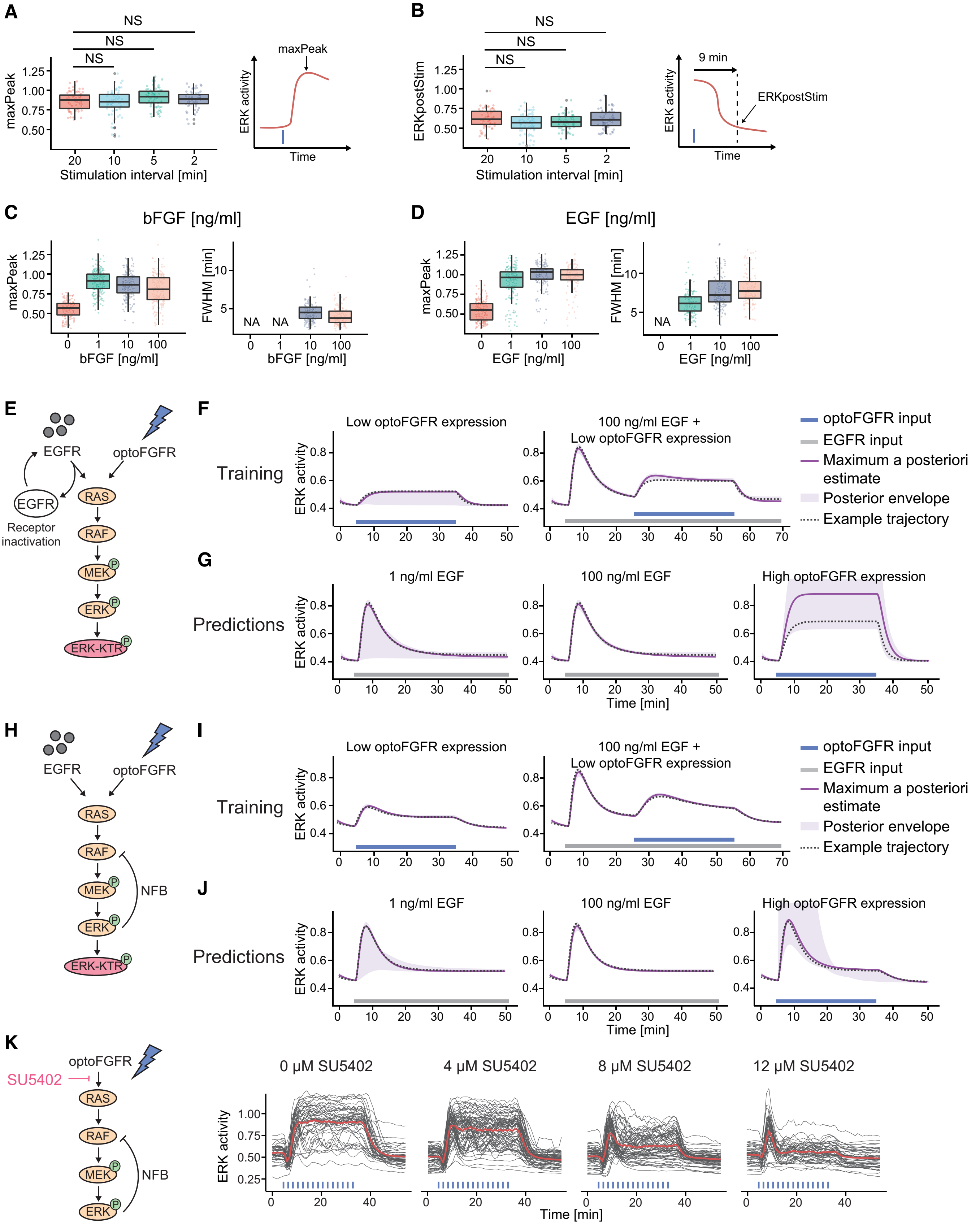

Figure EV1.

 © 2022 The Authors

Figure EV2. RNA interference screen reveals that ERK dynamics remain unaffected in response to perturbation of most MAPK signaling nodes.

A mRNA level quantification (RT–qPCR) of cells transfected with different concentrations of siRNA targeting *ERK1*, *ERK2*, *MEK1*, and *MEK2*. Data were normalized to the amount of *GAPDH* mRNA and relatively expressed to the corresponding mRNA in non-transfected cells.

B Western blot analysis of cells transfected with 10 nM of siRNA against the different *MEKs* and *RAFs* isoforms, *SOS1*, *GRB2*, *and RSK2*. Western blot analysis of cells transfected with the *ERKs* isoforms is shown in Fig 4C. Remaining protein levels were quantified by normalizing the amount of protein of interest (P.O.I) to the amount of GAPDH or actin protein and shown relatively to the corresponding protein level in the CTRL (individual values are displayed by dots (N = 2 replicates, apart from *RSK2* were N = 3), bar plots indicate the mean).

C, D Single-cell ERK responses under the different RNAi perturbations (sustained optoFGFR input, D = 18 mJ/cm^2, N = 40 cells randomly selected from low (C) and high (D) optoFGFR expression, out of at least 212 cells per perturbation from three technical replicates).

© 2022 The Authors

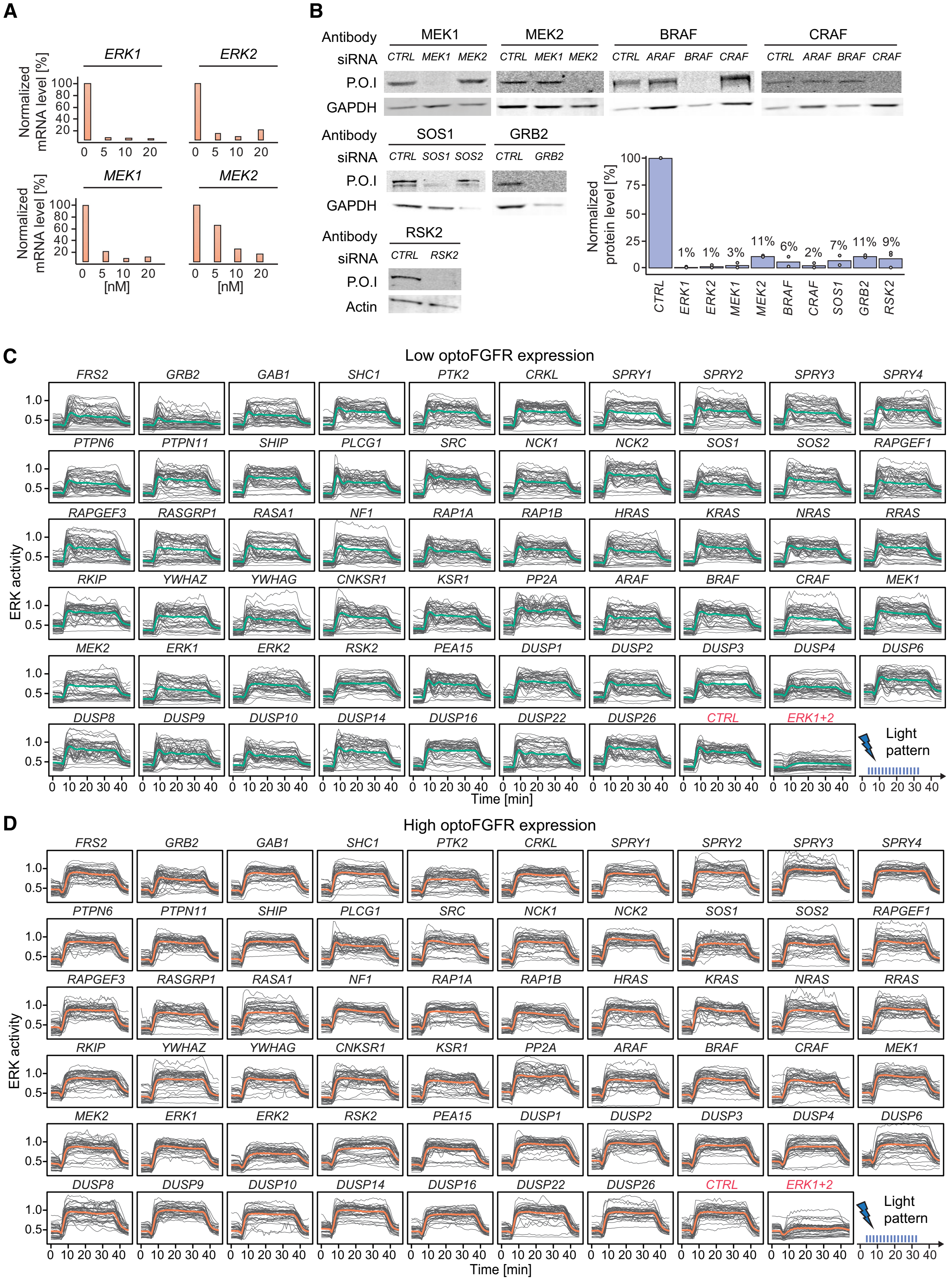

Figure EV2.

© 2022 The Authors

Figure EV3. Investigation of penetrant siRNA ERK dynamic phenotypes.

A Violin plot distributions of the baseline, maxPeak, and ERKpostStim of single-cell ERK responses under sustained high optoFGFR input (D = 18 mJ/cm^2, N_{min} = 126 cells with high optoFGFR expression per treatment, from three technical replicates). Boxes indicate the upper and lower quartiles, the central bands indicate the median, and whiskers extend to individuals up to 1.5 interquartile away from the median. Statistical analysis was done using a Wilcoxon test comparing each treatment to the control (*< 0.05, **< 0.005, ***< 0.0005, ****< 0.00005, FDR *P*-value correction method).

B Average ERK responses during a selected time window (dashed line, left panel) for selected siPOOLs affecting ERK adaptation (ERKpostStim in Fig 4D) (sustained optoFGFR input, D = 18 mJ/cm^2, N_{min} = 400 cells per condition from 3 technical replicates).

C CODEX was trained to recognize single-cell ERK responses evoked by sustained optoFGFR input (D = 18 mJ/cm^2) under the different RNAi perturbations (N_{min} = 212 cells per perturbation from 3 technical replicates).

D tSNE projection of the CNN features from CODEX trained on the 10 perturbations for which the classification accuracy on the validation set was the highest when trained on all perturbations (Appendix Table S4, see Materials and Methods), together with the non-targeting siRNA (*CTRL*).

E Single-cell ERK trajectories under 1 ng/ml sustained EGF input (added at t = 5 min) for selected RNAi perturbations (N = 50 cells per condition).

F Hierarchical clustering (Euclidean distance and Ward linkage) of single-cell ERK trajectories shown in (E) (N_{CTRL} = 300 cells, N_{ERK1+2} = 270 cells, N_{ERK2} = 320 cells, N_{CRAF} = 240 cells, N_{RSK2} = 340 cells). The number of clusters was empirically defined to resolve the different ERK dynamics. Average ERK responses per cluster are displayed on the right.

G Proportion of single-cell ERK trajectories per cluster shown in (F).

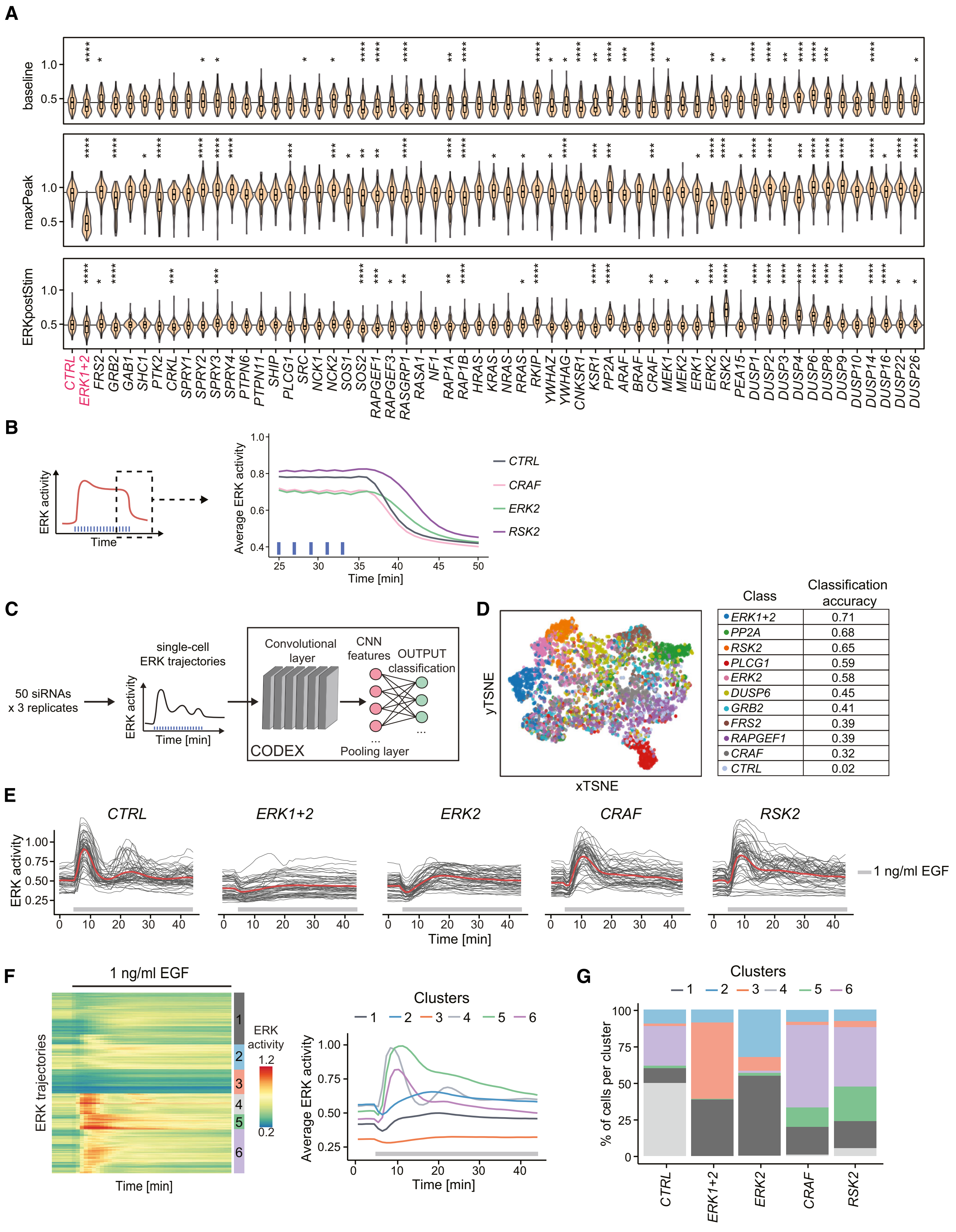

Figure EV3.

 © 2022 The Authors

Figure EV4. Direct optogenetic activation of RAS highlights different ERK dynamics phenotypes than optoFGFR input.

A Quantification of the maxPeak of ERK dynamics evoked by transient optoSOS input using different light doses (color code) and different numbers of 100 ms pulses (x-axis) repeated every 20 s (N_{min} = 136 cells from one replicate).

B Hierarchical clustering (Maximum distance and Ward D2 linkage) of ERK responses under 2-min interval optoSOS input shown in Fig 5C (D = 0.6 J/cm^2, N = 90 cells). The number of clusters was empirically defined to resolve the different ERK amplitudes. Average ERK responses per cluster are displayed on the right.

C Quantification of the baseline of single-cell ERK responses under sustained optoFGFR (Fig 2F, D = 18 mJ/cm^2) and optoSOS (Fig 5D, D = 0.6 J/cm^2) input for low or high expression of each optogenetic system (N = 40 cells per condition). Statistical analysis was done using a Wilcoxon test, comparing each condition to each other (N_{min} = 48 cells per condition, NS: non-significant, **< 0.005, FDR *P*-value correction method).

D, E ERK responses under RNAi perturbations targeting MAPK signaling nodes active below RAS (sustained optoSOS input, D = 0.6 J/cm^2, N = 40 cells from low (D) and high (E) optoSOS expressing cells for each perturbation, randomly selected out of at least 193 trajectories from three technical replicates).

F Violin plot distributions of the baseline, maxPeak and ERKpostStim of single-cell ERK responses under sustained high optoSOS input (D = 0.6 J/cm^2, N_{min} = 33 cells with high optoSOS expression per treatment, from three technical replicates). Statistical analysis was done using a Wilcoxon test comparing each treatment to the control (*< 0.05, **< 0.005, ***< 0.0005, ****< 0.00005, FDR *P*-value correction method).

G Average ERK responses during a selected time window (dashed line, upper panel) for selected siPOOLs affecting ERK adaptation (ERKpostStim in Fig 5F) (sustained optoSOS input, D = 0.6 J/cm^2, N_{min} = 270 cells per condition from three technical replicates).

Data information: In (A), (C), and (F), boxes indicate the upper and lower quartiles, the central bands indicate the median, and whiskers extend to individuals up to 1.5 interquartile away from the median.

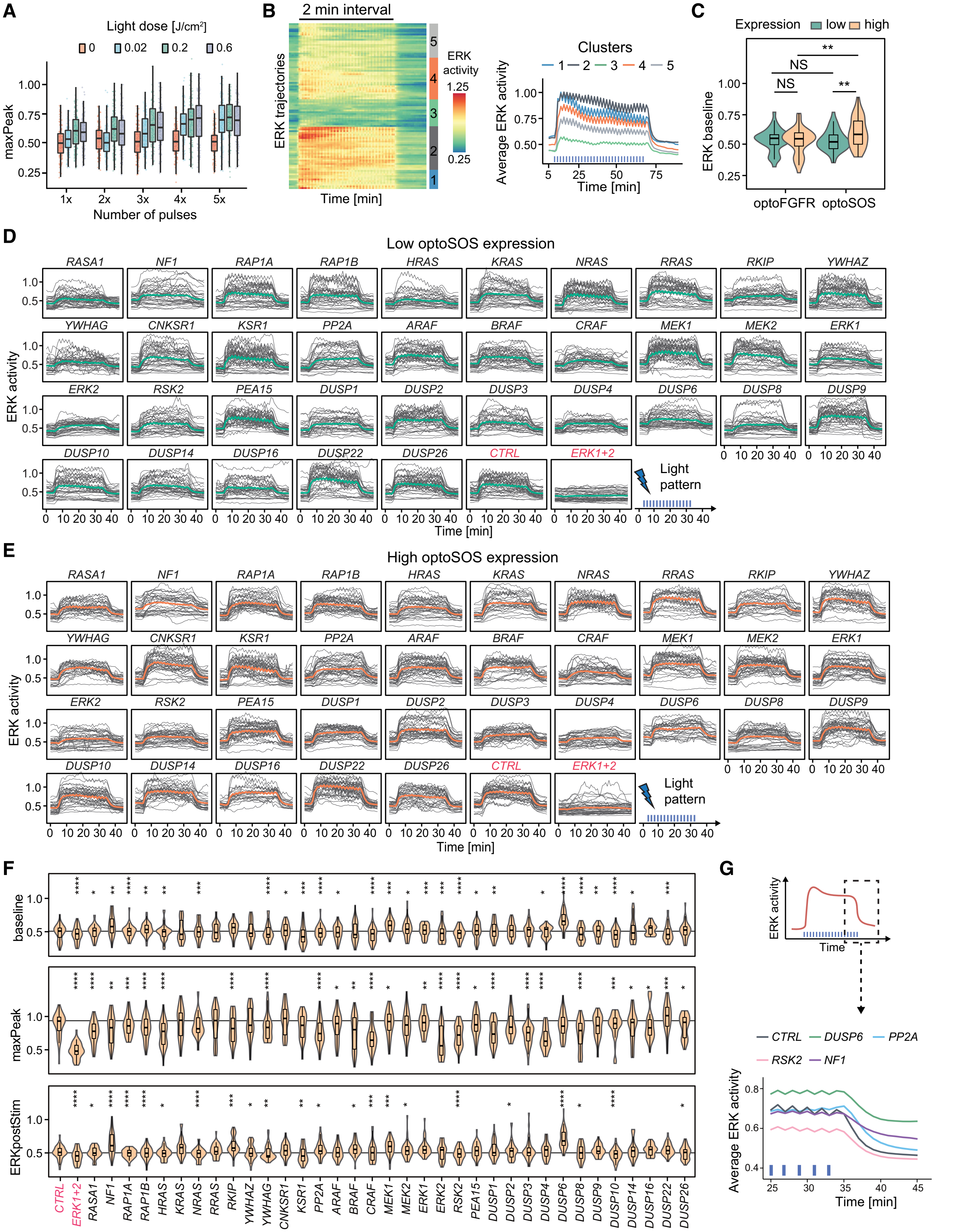

Figure EV4.

 © 2022 The Authors

Figure EV5. Perturbation of the RSK2-mediated NFB increases the efficiency of RAS, MEK, and ERK targeting drugs.

A Single-cell ERK trajectories from high optoFGFR or high optoSOS expressing cells treated with U0126 dose response (N = 40 cells per condition, randomly selected out of at least 200 cells from three technical replicates, sustained optoFGFR (D = 18 mJ/cm^2) or optoSOS (D = 0.6 J/cm^2) input).

B Single-cell ERK trajectories from high optoFGFR expressing cells treated with U0126 dose response for *CTRL* or *RSK2* KD cells (N = 40 cells per condition (apart from *RSK2* KD + 0 μM U0126 (32 cells)), from 2 technical replicates for *RSK2* KD and 1 replicate for *CTRL* KD, sustained optoFGFR input (D = 18 mJ/cm^2)). For (A) and (B), data for RAF709 and SCH772984 are available in supplementary material.

C Schematic representation of the optoFGFR system untreated (RSK2-mediated feedback dependent) or treated with an RSK2 inhibitor SL0101 (RSK2-mediated feedback independent) targeted with the B/CRAF (RAF709), the MEK (U0126) or the ERK (SCH772984) inhibitor.

D Single-cell ERK amplitudes from sustained high optoFGFR input (D = 18 mJ/cm^2) under different concentrations of the MAPK inhibitors, extracted at a fixed time point ($t_{\text{fixed optoFGFR}}$ = 15 min, N = 70 cells with high optoFGFR expression per condition (except for 100 μM SL0101 + 0 μM U0126 (36 cells)) randomly selected from 2 technical replicates).

E A Hill function was fit to the normalized mean ERK activity as shown in (D) ($N_{\min}$ = 36 cells per condition). Shaded area indicates the 95% CI and dashed lines the EC_{50}.

F Normalized standard deviation of ERK amplitudes shown in (D) ($N_{\min}$ = 36 cells per condition).

G U0126 dose response analysis in MCF10A WT cells stimulated with 10 ng/ml EGF at t = 30 min. ERK responses were extracted at a fixed time point following the steep rising phase (t_{fixed} = 50 min, N = 140 cells per condition).

H, I Average ERK responses of MCF10A WT cells with EGF stimulation (10 ng/ml EGF added at t = 30 min) (H) or of ErbB2 overexpressing (ErbB2^{over}) MCF10A cells without EGF stimulation (I) under no inhibitor, RSK (SL0101) inhibitor, MEK (U0126) inhibitor or a combination of both. Shaded areas indicate the 95% CI.

J, K tSNE projection of CODEX's CNN features per treatment from ERK activity of MCF10A WT cells (J) or MCF10A ErbB2^{over} cells (K) treated with 50 μM SL0101, 3 μM U0126 or a combination of both.

Data information: In (D) and (G), boxes indicate the upper and lower quartiles, the central bands indicate the median, and whiskers extend to individuals up to 1.5 interquartile away from the median.

© 2022 The Authors

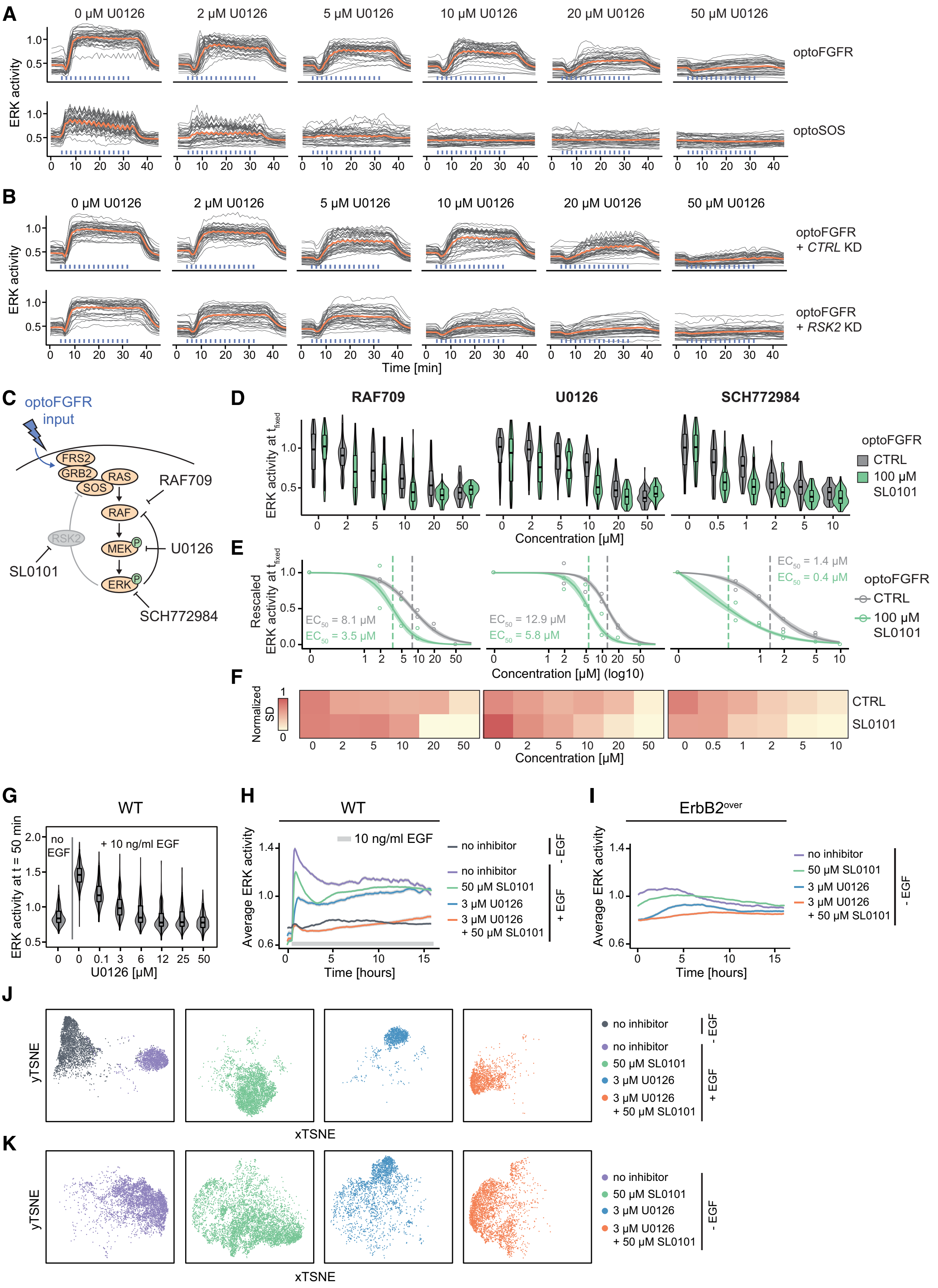

Figure EV5.

 © 2022 The Authors
