# Supplementary material for: Optogenetic actuator – ERK biosensor circuits identify MAPK network nodes that shape ERK dynamics
Source: Mol Syst Biol. 2022 Jun 13;18(6):e10670. doi: 10.15252/msb.202110670 (PMC9189677; doi:10.15252/msb.202110670)
Supplement: Supplementary file 3 — Movie EV1 [file MSB-18-e10670-s003.zip › Movie_EV1/README.rtf]

Movie EV1: ERK dynamics in response to a transient optoFGFR input. Cells stably expressing ERK-KTR-mRuby2, H2B-miRFP703 and optoFGFR-mCitrine were stimulated with a 470 nm light pulse (18 mJ/cm2) at t = 9 minutes (blue top band). ERK-KTR and H2B were acquired at 1-minute intervals with a 20x air objective. OptoFGFR was acquired at the end of the experiment (t = 40 minutes). ERK-KTR nuclear signal was segmented based on the H2B nuclear marker (green circle). ERK-KTR cytosolic signal was extracted in a 4-pixels ring around the nucleus. The ring was obtained by expanding the nuclear mask by 2-pixels (blue circle) to exclude the blurred edges of the nucleus and further expanding that new mask by 4 pixels in a threshold-based manner (pink circle). Single-cell ERK activity was then calculated as the cytosolic/nuclear ERK-KTR ratio. Scale bar: 50 μ.
