# Supplementary material for: Optogenetic actuator – ERK biosensor circuits identify MAPK network nodes that shape ERK dynamics
Source: Mol Syst Biol. 2022 Jun 13;18(6):e10670. doi: 10.15252/msb.202110670 (PMC9189677; doi:10.15252/msb.202110670)
Supplement: Supplementary file 4 — Movie EV2 [file MSB-18-e10670-s005.zip › Movie_EV2/README.rtf]

Movie EV2: OptoFGFR dimerization dynamics. Cell stably expressing optoFGFR-mScarlet was imaged at 20-second intervals with a 100x TIRF objective and stimulated with a 470 nm light pulse at t = 300 seconds (blue top band). The Ilastik pixel classification module was used to manually annotate and segment optoFGFR dimers/oligomers events without quantifying the endocytic vesicles (see Appendix Figure S1B). OptoFGFR dimerization was then quantified by computing the mean of pixel intensities from the binarized mask obtained with Ilastik using Fiji. Scale bar: 5 μ.
