# Supplementary material for: Optogenetic actuator – ERK biosensor circuits identify MAPK network nodes that shape ERK dynamics
Source: Mol Syst Biol. 2022 Jun 13;18(6):e10670. doi: 10.15252/msb.202110670 (PMC9189677; doi:10.15252/msb.202110670)
Supplement: Supplementary file 5 — Movie EV3 [file MSB-18-e10670-s001.zip › Movie_EV3/README.rtf]

Movie EV3: Different optoFGFR inputs trigger transient, oscillatory and sustained ERK dynamics. Cells stably expressing ERK-KTR-mRuby2, H2B-miRFP703 and optoFGFR-mCitrine were stimulated with 470 nm light pulse (18 mJ/cm2) at 2-minute intervals (blue top bands). ERK-KTR and H2B were acquired at 1-minute interval with a 20x air objective. OptoFGFR was acquired at the end of the experiment (t = 55 minutes). High optoFGFR level (cells 3, 4, and 5) lead to a sustained ERK activity, while low optoFGFR level (cells 1 and 2) lead to oscillatory ERK dynamics. Scale bar : 25 μ.
