# Supplementary material for: Optogenetic actuator – ERK biosensor circuits identify MAPK network nodes that shape ERK dynamics
Source: Mol Syst Biol. 2022 Jun 13;18(6):e10670. doi: 10.15252/msb.202110670 (PMC9189677; doi:10.15252/msb.202110670)
Supplement: Supplementary file 6 [file MSB-18-e10670-s004.pdf]

molecular systems biology

Optogenetic actuator - ERK biosensor circuits identify MAPK network nodes that shape ERK dynamics

Coralie Dessauges, Jan Mikelson, Maciej Dobrzyński, Marc-Antoine Jacques, Agne Frismantiene, Paolo Armando Gagliardi, Mustafa Khammash, and Olivier Pertz
DOI: 10.15252/msb.202110670

Corresponding author(s): Olivier Pertz (olivier.pertz@izb.unibe.ch)

Review Timeline:

Submission Date:	3rd Sep 21
Editorial Decision:	19th Oct 21
Revision Received:	17th Mar 22
Editorial Decision:	6th May 22
Revision Received:	16th May 22
Accepted:	17th May 22

Editor: Jingyi Hou

Transaction Report:

(Note: With the exception of the correction of typographical or spelling errors that could be a source of ambiguity, letters and reports are not edited. Depending on transfer agreements, referee reports obtained elsewhere may or may not be included in this compilation. Referee reports are anonymous unless the Referee chooses to sign their reports.)

Thank you for submitting your work to Molecular Systems Biology. We have now heard back from the three reviewers who agreed to evaluate your manuscript. As you will see from the reports below, the reviewers acknowledge the potential interest of the study. They raise, however, a series of concerns, which we would ask you to address in a major revision.

Without reiterating all the points raised in the reviews below, some of the more substantial issues are the following:

- Both Reviewers #1 and #3 mentioned that the definition of "robustness" seemed somewhat unclear, and they raised concerns about the data interpretation in this regard.
- The Reviewers shared overlapping concerns about the data supporting the RSK2 feedback. During our pre-decision cross-commenting process (in which the reviewers are given a chance to make additional comments, including on each other's reports), Reviewers #1 thought that "It seems that these issues can largely be addressed with a major revision of the text that emphasizes the technical points and presents the RSK2 story in a more reserved way", which was concurred by Reviewer # 1. Therefore, we would ask you to address this raised issue according to both reviewers' comments.

Other issues raised by the reviewers need to be satisfactorily addressed as well. As you may already know, our editorial policy allows in principle a single round of major revision, and it is therefore essential to provide responses to the reviewers' comments that are as complete as possible.

On a more editorial level, we would ask you to address the following issues:

REFEREE REPORTS

Reviewer #1:

In this work Pertz and colleagues establish a combination optogenetic and live cell reporter system to investigate ERK dynamics. RTK-RAS-ERK signalling is one of the most intensely studied pathways in cell biology, and certainly in the field of single cell signalling dynamics. This comes with the advantages that many tools have been developed (especially by the Pertz lab) to study this system, and there is extensive prior data on which to build models that can be tested.
And I have to say - much of this work is a real technical achievement. Combining these optogenetic tools with these sensors cannot be straightforward, and probably is not achievable by most labs.

With these tools in hand the authors repeatedly claim to be testing 'robustness' in different contexts - which in the work is very poorly defined. In fact, there is no insight here as to what makes cells/networks robust. Overall, I think the authors went overboard on interpreting the results of particular experiments in the light of robustness to perturbation. It is not particularly important to ask whether a particular signalling dynamic/motif (i.e. ERK oscillations) that occurs over a short time is robust to perturbation. It is more important to ask how the system as a whole (particular behaviours, the cell, etc...) is/isn't robust to perturbation - and this is not in the scope of this manuscript. Cells did not evolve the dynamics of the MAPK network to be robust. The dynamics of the MAPK network evolved to provide robustness of cell behaviour to flux. In particular the authors attempt to claim an arbitrarily sub-network is more/less robust to perturbation than another arbitrarily sub-network was extremely naïve.
Rather most of the data provides potential mechanistic insight into ERK dynamics in some particular cases - which in itself is interesting! The genetic screen is very elegant, and I think data like this could certainly be mined to provide new mechanisms of ERK regulation. That said - the authors appeared to be reluctant to go down this road.
Much of the insight comes in the form of testing whether previously established negative regulators - especially RSK2 - are involved in regulating ERK dynamics in this system. On one hand, I think this is very important to do. While numerous regulators and effectors of ERK have been identified, and some have been implicated in feedback, whether they are involved in this system is essential to figure out. The authors are making a reasonable decision to test previous models.
However, performing this work comes with a catch - proving the existence of different previously identified regulators/loops provides no novelty. I sympathize with the authors here. It was presumably a massive challenge just to show that RSK2 is indeed a (potential) negative regulator here - but this is not discovery.
Overall I would recommend some revision to make this more of a technical study of ERK dynamics and tools, with the RSK2 vignette being an important validation of the system (with the caveat I do have some major issues here). Even though nothing new was necessarily discovered and validation, I still think its such a technical accomplishment that the readers of Molecular Systems Biology could find it of interest. I would recommend that some further experiments might still be required to conclusively establish some concept. Moreover, that there is no discussion of robustness in the text.

Specific points:
"Subsequent application of the optoFGFR input yielded identical sustained ERK responses than in non-pre-stimulated cells with high optoFGFR expression. However, EGF pre-stimulation reduced the occurrence of optoFGFR-evoked oscillatory ERK responses in low optoFGFR expressing cells.... Thus, our results suggest that strong EGFR input pre-activates the same negative feedback responsible for the oscillatory ERK dynamics in response to optoFGFR inputs."
This seems unclear to me. How did the authors draw this conclusion? There are fewer oscillatory cells? Or the oscillations themselves are dampened? It looks to me as if there is still a subset of cells in the optoFGFR that are oscillating after EGF stimulation.
It is an important point - because as the authors write, this would suggest that the negative feedback loops that act downstream of FGFR can be affected by EGF. Is this a bit surprising? I am not sure how the 'within' pathway negative feedbacks would be affected differently by EGF + FGF vs just FGF.
"Oscillatory ERK dynamics has been shown to emerge from the presence of negative feedbacks (Kholodenko, 2000)."
Has it? I think the paper shows that it 'could' result from this. I think its too strong to immediately say that this is happening in these cells.
"To provide intuition about the MAPK network circuitries leading to different ERK dynamics in response to optoFGFR and EGFR inputs, as well as the origin of the oscillatory behavior, we built a mathematical model consisting of the RAS GTPase and the three-tiered RAF/MEK/ERK network (Figure 3D, Appendix Table S1)."
This modelling (and the results) all seem sensible. I am not sure it really provided much insight into the interesting questions that have been raised by this point - i.e. such as the source of the negative feedback, and the reason that EGF pre-stimulation appears to have some effect on FGF.
"We show that perturbation of this feedback can weaken the MAPK network in an oncogenic signaling model, allowing to sensitize ERK activation to MEK inhibition, and to robustly shut down ERK activity in all the cells of a population. Thus, identification of the MAPK feedback circuitry provides a rationale to weaken the MAPK network for potent pharmacological inhibition of ERK in cancer.

What does 'weaken the network' mean? Networks aren't weak or strong.
"the RAS/ERK subnetwork is less resilient against node perturbations than the full MAPK network."
I really find this statement silly. There is really no such thing as the RAS/ERK subnetwork in cell. We use 'subnetworks' as simplified constructs for analysis, presentation, and interpretation. The cell, and the evolutionary processes that drive robusteness, do not care about the differences between one sub-network and another.
"The RSK2 negative feedback confers robustness to the MAPK network against 389 MEK inhibition"
Again - the cell did not evolve the MAPK network to be robust. The dynamics of the MAPK network evolved to provide robustness of cell behaviour to flux.
In addition...
The authors combine optoFGFR/optoSOS and RSK2KDto test the hypothesis that RSK2 mediates a negative feedback loop activated by ERK1/2 which inhibits SOS in this system to promote oscillations. Notably, this is based on previous studies which have implicated RSK2 as a negative regulator of SOS downstream of ERK. Lack of novelty aside, I think it is important to show whether such feedbacks are occurring in this particular system.
It took quite a bit of thinking to get my head around what you might expect, and once I did - I think the results don't quite meet

my theoretical predictions. The experiments are well done but it is not an obvious effect of RSK2 KD -, and the statistics seems very borderline to me (Figure 6H).
I also don't understand why the RSK2 KD doesn't increase ERK signalling (max increase or rate of activation) in the absence of MEK inhibition?
Can they firmly confirm the existence of this feedback loop? I am not so sure. Is it likely there? Probably? But it seems more experimental proof should be required here. Critically, there could be other explanation for this data.
That said, I am not at all convinced by the differential analysis of RSK2KD in optoFGFR vs optoSOS. While certainly the data suggests RSK2 could be involved in negative feedback, I do not see how they decided to put it at the level of SOS based on these experiments. Could it not just be that the signalling downstream of FGFR vs SOS is different in magnitude and/or type (number of effectors which interact with ERK signalling up- and down-stream?)
I think this is a major issue. Despite the fact it probably does exist, I don't think the authors have actually proven it here.

Reviewer #2:

This manuscript describes a study on single cell ERK dynamics, specifically how a host of proteins that when knocked down influence the dynamics as controlled by an optogenetic FGFR system. The main impactful claim of the work is that ERK dynamics are robust to inhibition, unless a negative feedback loop they found involving RSK (already known) is targeted along with direct inhibition of the ERK pathway (MEK inhibition). This idea that the negative feedback loops lead to robustness of the ERK pathway is not new (as cited by the authors), although to my knowledge nobody has shown specifically that targeting the RSK negative feedback loop is key. That being said, I found the evidence for this claim to be weak. First, the inhibitor doses being used were very high, so the possibility that off-target effects of the RSK and MEK inhibitors are leading to the varying robustness observations are high. Moreover, the MEK inhibitor being used, U0126, is an older one that has less specificity than newer generation ones. In Fig. 6, the choice of time point at which ERK activity effects of the inhibitor is evaluated may have a significant impact on the claim of decreased robustness. RSK knockdown only decreases the IC50 for the MEK inhibitor for ERK activity at this fixed time point by two-fold---what is the uncertainty in that estimate? And how does the RSK knockdown (or inhibition) affect cell viability, in more standard drug response assays? These are significant concerns that cast doubt upon the main impactful claim of the paper.
That being said, the expansive knockdown data set on how various protein levels influence ERK dynamics I thought was very useful and informative, and would be a welcome addition to the literature on the topic. The method for selecting which proteins to knockdown was interesting and useful.

Reviewer #3:

This manuscript from Dessauges and colleagues presents a deep analysis of ERK activity dynamics, using a combination of optogenetic tools, siRNA-based knockdowns, and various quantitative analyses. A major conclusion is that the dynamics of growth factor receptor-stimulated ERK activity are largely unperturbed by knockdown of a majority of the pathway components. A parallel analysis with a second optogenetic stimulation that operates downstream of the receptor is used to demonstrate that this robustness arises from feedback to the receptor level, rather than feedback operating within the Ras-Raf-MEK-ERK cascade. The authors then look further into the specific mechanism of feedback from ERK to receptor via RSK2, and attempt to make the case that targeting this negative feedback route can reduce the robustness of the ERK dynamics and increase the efficacy of MEK inhibition as a pathway-targeting strategy.
The first five figures of this work are extremely impressive - a technical tour de force that sets a new standard for the entire field. Compared to the western blot-based studies that still dominate the signaling literature, this paper demonstrates nothing short of a quantum leap in the ability to collect carefully validated, highly quantitative data on a large scale, producing a dataset with great power to better understand growth factor signaling at the systems level. Every step in the process is carefully validated and clearly presented. The large dataset reveals the single-cell intricacies of ERK signaling in much greater depth than any other work and will undoubtedly power many modeling studies to come. We are extremely enthusiastic to see this data and analysis published quickly.

We had more reservations about the last two figures of the paper, and the conclusions drawn concerning the effect of RSK2-mediated feedback on the robustness of the pathway. As detailed further in the points below, the concept of robustness as it applies in this case is not well defined, and a number of the individual experiments don't provide strong support for the conclusions that are being made. We found this section much harder to get through without stumbling on various inconsistencies, and we think it needs some revision to better justify the overall conclusions.

Major points
1. The concept of "robustness" as it applies to ERK outputs is not clearly defined, especially in the section from 388-409. It is

difficult to tell whether robustness is being equated with the decrease in heterogeneous ERK behavior, or a lower IC50 for MEK inhibition. There are important differences between these characteristics, and we were not sure whether the focus is meant to be on biological robustness (where diversity of cell phenotypes may be advantageous) or on pharmacological robustness (sensitivity to inhibition). Some clarification is needed here. Also, might it be a better representation of robustness, from the standpoint of pathway output, to look at the shallower dose response relationship seen in optoSOS rather than a simple change in IC50?

2. The differences between optoSOS and optoFGFR in MEK inhibition are clear (Fig. 6C-D), but the effects of RSK2 knockdown (Fig. 6G-H), and inhibition (EV5C-D) are substantially weaker. There are other known negative feedbacks affecting receptor internalization (e.g. Sprouty, etc.) which likely account for the difference, but the narrative seems to focus on RSK2 over these other possibilities in a way that doesn't seem fitting with the relatively modest experimental results.

3. The effects of RSK2 feedback perturbation could be better understood if they were more connected to the modeling. The fact that inhibiting RSK2-based negative feedback both decreases amplitude and leads to higher sensitivity to MEK inhibition is somewhat counterintuitive. We assume this effect arises because of differences in "thresholds" of MEK/ERK activity needed to activate the two different feedbacks? It seems that the models could be used more here to gain some intuition into the observed RSK2 inhibition results. Alternatively, the authors' understanding of what is happening to decrease ERK amplitude/increase MEK inhibition sensitivity in RSK2-knockdown/inhibited cells could be more clearly explained.

4. The section from line 440 to 460 relies heavily on interpreting the dispersion of cells within tSNE plots as "compact" or spread. It is now widely appreciated that tSNE plots can wildly distort distances between points, and this ambiguity makes it unclear how useful the dispersion of points in these visualizations can be in representing the heterogeneity of cells.

5. The cell cycle experiment in Figure 7 is difficult to interpret. Since RSK has ERK-independent effect, it is difficult to know how much of combined treatment's effect can be attributed to reduced ERK dynamics rather than combined effects on cell cycle mediators downstream of ERK. While I understand the objective to demonstrate an effect at the cellular phenotype level, I don't think this result clearly adds to the intended point about targeting feedback to sensitize the ERK pathway.

6. The overall dataset makes it clear that the ERK network is quite resistant to perturbations of single genes. However, the strong effect of an ERK1+ERK2 knockdown also hints that the combined perturbations of the same node can cause quite large changes. This raises the question of how much of the apparent robustness is really due to feedback regulation, rather than simply to gene redundancy. While it seems most reasonable to save an in depth experimental analysis of combined knockdowns for a later study, this point does warrant some discussion. Furthermore, there remains the possibility that some dosage compensation of expression levels could occur; although this seems unlikely given the timescale of the knockdowns, it deserves some mention as a caveat.

Minor points

On line 429, is "potentiate" intended rather than "potential"?

Lines 355-366 seem to have a redundant "light pulses".

Line 399 - U0126 "misspelled".

The color scheme in figure 7J and K is confusing and seems like it may be mislabeled. The ordering of colors in the stacked bars doesn't match their numerical order (why is 4 on top of 5?). The similarities of colors used in the heatmap and those used to represent the different clusters is also a bit disorienting.

Not much mention is made of the effects of knockdowns on basal state, other than DUSP6. It would be interesting and helpful to include a supplemental plot similar to Fig. 4D comparing changes in basal ERK activity.

General comments

We would like to thank all the reviewers for their comments and for giving us the opportunity to submit a revised version of our manuscript. It was a challenge to extensively present the large and complex dataset we generated in a concise way. We believe that the reviewers' comments have enabled us to show our data more clearly. We agree that some concepts and findings could have been better described. We have now revised our manuscript to answer the reviewers concerns with the following major modifications:

- As suggested from the comments of the reviewers, we modified the title of the manuscript to remove the concept of "MAPK robustness" and put the focus on the technological approach we developed to investigate the regulation of ERK dynamics.

- The different reviewers have made comments that our use of the term robustness was unclear. The difficulty is that the term robustness has different meanings in different contexts. In this work, we used the term robustness to describe the ability of the MAPK network to produce consistent ERK dynamics (in terms of amplitude and shape) in presence of strong node perturbations. We solve this problem by giving a clear definition of what we mean by signaling robustness in the introduction. Please note that this term had already been used to describe the limited effect of the MEK inhibitor U0126 on ERK response reduction by Sturm et al. (Sturm et al. 2010). It was also used by Fritsche-Guenther et al. to describe how the MAPK network can produce reliable ERK responses despite strong perturbation of its network components (Fritsche-Guenther et al. 2011).

- We realized that the reasons why we selected the *RSK2* KD phenotype as a worthwhile target to follow up in the last part of the paper was not clear to the reviewers. We worked on this to better guide the reader to this conclusion by providing a more detailed characterization of the phenotypes of our siRNA screens:

    - We now present a clearer rationale as to why we used sustained optoFGFR inputs to investigate the role of the 50 MAPK signaling nodes in ERK dynamics.
    - We now show a more complete feature-extraction based analysis of the perturbation screen, both for optoFGFR (Figure 4D, EV3A) and optoSOS (Figure 5F, EV4F) systems. The new panels provide an overview and statistical significance of the effects of the perturbations on ERK baseline, amplitude and adaptation (rather than amplitude only in the previous manuscript). These data further highlight a role for RSK2 in ERK adaptation in the optoFGFR system, but not in the optoSOS system. Adding the *RSK2* KD in the optoSOS screen reveals a decrease in ERK amplitude (as already seen in the optoFGFR screen), suggesting that, in addition to its role in the ERK-RSK2-SOS feedback, RSK2 also regulates nodes downstream of RAS.
    - We have moved the quantification of oscillatory ERK trajectories per RNAi perturbations from the supplementary material to the main figure to make a stronger statement about the role of RSK2 in the regulation of oscillatory ERK dynamics (Figure 4F).

- While not requested by the reviewers, we have conducted 3 siRNA screen replicates of the optoSOS system which improves the statistical relevance of our results (Figure EV4D,E). This has led to slightly different results than in the first draft of the manuscript. For example, *PP2A* and *DUSPs* phosphatases KD have yielded different phenotypes (Figure EV4F). Importantly, the conclusion about increased sensitivity to perturbations of the optoSOS versus the optoFGFR system remains valid.

- While not requested by the reviewers, we have added the Western blot quantification of *RSK2* KD efficiency to further validate our result (Figure EV2B).

- To answer concerns regarding our previous choice of MEK inhibitor (U0126) and further investigate the role of RSK2 in MAPK signaling robustness, we have added data from two newer-generation MAPK inhibitors: a B/CRAF inhibitor (RAF709) and an ERK inhibitor (SCH772984). These additional data strengthen the idea that the RSK2 feedback is implicated in MAPK signaling robustness. To analyze these data, we quantified how the single versus combined perturbations affect ERK amplitudes (Figure 6B,F,EV5D), the EC_{50} (Figure 6C,G,EV5E) and the distribution of ERK amplitude inhibition across cells (Figure 6D,H,EV5F). Additionally, we feel that we had failed to convey that we do not only want to document that combined perturbations act synergistically in inhibiting ERK, but that they also lead to more homogeneous inhibition in the cell population. This can be seen by looking at the ERK amplitude distributions (Figure 6B,F,EV5D), which is also quantified by the standard deviation of these distributions (Figure 6D,H,EV5F). We have more clearly emphasized this fact in the paper.

- We have removed the analysis of cell cycle entry from Figure 7. As pointed out by reviewer 3, RSK2 is known to have ERK-independent effect on cell cycle regulation, which makes a clear interpretation of our results difficult.

- Following the comments of reviewer 3 on the interpretation of the tSNE plots presented in figure 7, we now have used a fully automated approach to cluster the CNN features and extract representative single-cell trajectories for each cluster. This approach illustrates the heterogeneity in ERK dynamics in response to different treatments without any human bias.

- We have revised our discussion to integrate our new experimental data and their analysis, as well as to address the issues raised by the reviewers. We now discuss more carefully the fact that the RSK2 feedback plays a role in MAPK signaling robustness but is probably not the only factor involved. We have also more clearly discussed the limitations of our approach.

Modified parts of the manuscript are highlighted in blue. We have copied below the comments of the reviewers in black font and answered them point by point in blue font.

Reviewer #1:

In this work Pertz and colleagues establish a combination optogenetic and live cell reporter system to investigate ERK dynamics. RTK-RAS-ERK signalling is one of the most intensely studied pathways in cell biology, and certainly in the field of single cell signalling dynamics. This comes with the advantages that many tools have been developed (especially by the Pertz lab) to study this system, and there is extensive prior data on which to build models that can be tested.

And I have to say - much of this work is a real technical achievement. Combining these optogenetic tools with these sensors cannot be straightforward, and probably is not achievable by most labs.

With these tools in hand the authors repeatedly claim to be testing 'robustness' in different contexts - which in the work is very poorly defined. In fact, there is no insight here as to what makes cells/networks robust. Overall, I think the authors went overboard on interpreting the results of particular experiments in the light of robustness to perturbation. It is not particularly important to ask whether a particular signalling dynamic/motif (i.e. ERK oscillations) that occurs over a short time is robust to perturbation. It is more important to ask how the system as a whole (particular behaviours, the cell, etc...) is/isn't robust to perturbation - and this is not in the scope of this manuscript. Cells did not evolve the dynamics of the MAPK network to be robust. The dynamics of the MAPK network evolved to provide robustness of cell behaviour to flux. In particular the authors attempt to claim an arbitrarily sub-network is more/less robust to perturbation than another arbitrarily sub-network was extremely naïve.

We thank the reviewer for his/her positive comments about our new systems to study ERK dynamics. This reviewer, as well as the others have made the comment that our use of the term robustness was misleading. With the term signaling robustness, we aimed to describe the ability of the MAPK network to produce consistent ERK dynamics (in terms of amplitude and shape) in presence of strong node perturbations. We now clearly explain our definition of signaling robustness in the introduction. We have also added data from two other MAPK inhibitors targeting B/CRAF (RAF709) or ERK (SCH772984) to the previously shown MEK inhibitor data (Figure 6, EV5). The results further strengthen the concept of MAPK signaling robustness, as well as the role of the RSK2-mediated NFB in this process. While we agree that it is important to study how a cellular system or behavior is robust against perturbations, in our paper we specifically focused on the MAPK network responses.

We thank the reviewer for letting us know that the term “subnetwork” was incorrect. We now refer to the RAS/RAF/MEK/ERK network when referring to the network activated with the optoSOS ERK dynamics are triggered from RAS using optoSOS, bypassing some signaling nodes.

Rather most of the data provides potential mechanistic insight into ERK dynamics in some particular cases - which in itself is interesting! The genetic screen is very elegant, and I think data like this could certainly be mined to provide new mechanisms of ERK regulation. That said - the authors appeared to be reluctant to go down this road.

Much of the insight comes in the form of testing whether previously established negative regulators - especially RSK2 - are involved in regulating ERK dynamics in this system. On one

hand, I think this is very important to do. While numerous regulators and effectors of ERK have been identified, and some have been implicated in feedback, whether they are involved in this system is essential to figure out. The authors are making a reasonable decision to test previous models. However, performing this work comes with a catch - proving the existence of different previously identified regulators/loops provides no novelty. I sympathize with the authors here. It was presumably a massive challenge just to show that RSK2 is indeed a (potential) negative regulator here - but this is not discovery.

We focused on the previously described RSK2 feedback because it led to one of the most robust phenotypes observed in our siRNA screen. We would like to underline that, while we initially expected to observe many interesting ERK dynamics phenotypes, only a limited set of perturbations affected ERK dynamics (Figure EV2C,D). The ability of the MAPK network to function efficiently under the perturbation of most of its nodes limited the study of new mechanistic insights. Striking phenotypes, such as PP2A or DUSP6, that lead to increased ERK amplitude and baseline (Figure 4D, EV3A), provided important sanity checks about the validity of our experimental system, but they did not provide new mechanistic insides into the MAPK signaling regulation.

We now provide a better explanation of the reasons why we focused on RSK2 (Figure 4D-F). It is true that the RSK2 feedback has already been described before, but we believe that the novelty of our results lays in the fact that nobody has ever shown experimentally that this feedback shapes ERK response duration (Figure 4D,EV3A,B) and oscillatory behavior (Figure 4F), might work simultaneously with the classic ERK to RAF negative feedback (Figure 4F) and is important to produce consistent ERK output despite node perturbation (Figure 6,7,EV5). The reviewer is right to assume that these conclusions were not straightforward – even if the RSK2 phenotype is one of the most penetrant, it remained difficult to spot, especially in a context in which we had to visually inspect 1000s of trajectories. Identifying this phenotype required several analytical approaches (Feature analysis, CODEX and quantification of oscillations - Figure 4D-F). To be convinced about this phenotype, we also evaluated *RSK2* KD in response to an EGF input (Figure EV3E-G).

Overall I would recommend some revision to make this more of a technical study of ERK dynamics and tools, with the RSK2 vignette being an important validation of the system (with the caveat I do have some major issues here). Even though nothing new was necessarily discovered and validation, I still think its such a technical accomplishment that the readers of Molecular Systems Biology could find it of interest. I would recommend that some further experiments might still be required to conclusively establish some concept. Moreover, that there is no discussion of robustness in the text.

By modifying the text and several figures, we hope to have now clarified why we focused on the RSK2-mediated feedback and why this brings important new insights into the MAPK signaling network regulation. Additionally, we now provide new data to show that that inhibition of the RSK2-mediated feedback sensitizes ERK inhibition not only to MEK inhibition, but also to RAF and ERK inhibition (Figure 6, EV5). These additional data give more strength to our finding about the role of the RSK2-mediated feedback in producing consistent ERK outputs despite node perturbation. This observation was validated in the context of WT and in ErbB2 overexpressing MCF10A systems, which provides relevance to

cancer. We agree that some of our statements regarding the role of RSK2 in MAPK network insensitivity to node perturbation were too strong. We now discuss more carefully the fact that the RSK2-mediated feedback is an important player, but probably not the only one. We also more carefully define what we mean by robustness in the context of this work.

Specific points:

"Subsequent application of the optoFGFR input yielded identical sustained ERK responses than in non-pre-stimulated cells with high optoFGFR expression. However, EGF pre-stimulation reduced the occurrence of optoFGFR-evoked oscillatory ERK responses in low optoFGFR expressing cells.... Thus, our results suggest that strong EGFR input pre-activates the same negative feedback responsible for the oscillatory ERK dynamics in response to optoFGFR inputs."
This seems unclear to me. How did the authors draw this conclusion? There are fewer oscillatory cells? Or the oscillations themselves are dampened? It looks to me as if there is still a subset of cells in the optoFGFR that are oscillating after EGF stimulation.
It is an important point - because as the authors write, this would suggest that the negative feedback loops that act downstream of FGFR can be affected by EGF. Is this a bit surprising? I am not sure how the 'within' pathway negative feedbacks would be affected differently by EGF + FGF vs just FGF.

The reviewer is right to say that there is a small set of cells still capable to perform some oscillations in response to optoFGFR inputs following the EGFR input. However, we did not observe a robust and synchronous oscillatory behavior as in low optoFGFR expressing cells in response to optoFGFR input only (Figure 3C). Our claim is that the same “within” NFB is activated by both EGFR and optoFGFR input. Thus, in the case of the EGFR + optoFGFR stimulations, pre-activation with sustained EGF pre-activates this NFB so that when the optoFGFR input is applied, the NFB has already reached a steady state, explaining the blunted oscillatory responses. When cells are not pre-stimulated with EGF, the NFB started being activated by the optoFGFR input, which results in ERK oscillatory behaviors until a steady state is reached. This explanation was supported by our modeling efforts (Figure 3D-G, Figure EV1E-J).
The reviewer might find this set of experiments and modeling trivial, but to us, these experiments provided essential sanity checks that validated our experimental system. Importantly, optoFGFR lacks the ectodomain present in the endogenous FGFR. This ectodomain, through a set of complex interactions, is important for shaping ERK dynamics, as we and others have shown before (Blum et al. 2019; Kanodia et al. 2014). Therefore, optoFGFR must be considered as a prototype RTK, which does not behave the same as the endogenous FGFR.

"Oscillatory ERK dynamics has been shown to emerge from the presence of negative feedbacks (Kholodenko, 2000)."
Has it? I think the paper shows that it 'could' result from this. I think its too strong to immediately say that this is happening in these cells.

We thank the reviewer for pointing this out. Indeed, the specific ERK dynamics we observe could emerge from different MAPK network topologies. We have now modified the statement and reference to be more cautious when explaining our hypothesis. We however believe that the presence of negative feedback(s) is a reasonable assumption given that (1) combination of EGF + optoFGFR inputs resulted in a strong dampening of the oscillations (Figure 3C), and (2) KD of *ERK2*, *CRAF* and *RSK2*, which are part of two NFBs previously mapped, strongly dampened ERK oscillatory behavior (Figure 4F).

"To provide intuition about the MAPK network circuitries leading to different ERK dynamics in response to optoFGFR and EGFR inputs, as well as the origin of the oscillatory behavior, we built a mathematical model consisting of the RAS GTPase and the three-tiered RAF/MEK/ERK network (Figure 3D, Appendix Table S1)."
This modelling (and the results) all seem sensible. I am not sure it really provided much insight into the interesting questions that have been raised by this point - i.e. such as the source of the negative feedback, and the reason that EGF pre-stimulation appears to have some effect on FGF.

The purpose of our modeling effort was to characterize our prototype optoFGFR system. Our model provides coarse-grained understanding of the mechanism responsible for our experimental results, which was not intuitive from visual examination of ERK dynamics. The main insights are that:

1. The difference between the sustained response to high optoFGFR input and the transient adaptive response to high EGFR input could be explained by difference in receptor regulations. This was further supported by the fact that dose response inhibition of the optoFGFR led to a transient adaptive response similar to the transient response obtained with EGFR input (Figure EV1K) and by previous works showing that the strong endocytosis of EGFR plays a major role in the transient ERK response (Kiyatkin et al. 2020; Gerosa et al. 2020).
2. The observed oscillatory ERK dynamics could be explained by the presence of a NFB, which we placed for the modeling purposes between ERK and RAF based on previous works (Santos et al. 2007; Kholodenko et al. 2010; Fritsche-Guenther et al. 2011; Blum et al. 2019).

Even if this might sound trivial to the reviewer, these results suggest that our prototype RTK activates a NFB downstream of an endogenous RTK, and therefore can be used to learn about ERK dynamics regulation. In our model, we placed this NFB at the level of RAF. However, at that state in the paper, we do not claim that the NFB responsible for the oscillations must be at this level. Indeed, our model does not exclude that other network topologies might be able to produce the observed ERK dynamics. We now state this more clearly in the manuscript.

We show that perturbation of this feedback can weaken the MAPK network in an oncogenic signaling model, allowing to sensitize ERK activation to MEK inhibition, and to robustly shut down ERK activity in all the cells of a population. Thus, identification of the MAPK feedback circuitry provides a rationale to weaken the MAPK network for potent pharmacological inhibition of ERK in cancer.
What does 'weaken the network' mean? Networks aren't weak or strong.

We thank the reviewer for pointing that our terminology was not adequate. We now removed the concept of “weak” or “strong” network.

"the RAS/ERK subnetwork is less resilient against node perturbations than the full MAPK network."
I really find this statement silly. There is really no such thing as the RAS/ERK subnetwork in cell. We use 'subnetworks' as simplified constructs for analysis, presentation, and interpretation. The cell, and the evolutionary processes that drive robustness, do not care about the differences between one sub-network and another.

We thank the reviewer for pointing that our terminology was not adequate. We have removed the term “subnetwork” and refer to the RAS/RAF/MEK/ERK part of the network when describing the network activated from RAS.

"The RSK2 negative feedback confers robustness to the MAPK network against MEK inhibition"
Again - the cell did not evolve the MAPK network to be robust. The dynamics of the MAPK network evolved to provide robustness of cell behaviour to flux.

Again, we thank the reviewer for pointing that our terminology was not adequate. We have now reformulated sentences that might convey a message of evolution.

In addition...
The authors combine optoFGFR/optoSOS and RSK2KD to test the hypothesis that RSK2 mediates a negative feedback loop activated by ERK1/2 which inhibits SOS in this system to promote oscillations. Notably, this is based on previous studies which have implicated RSK2 as a negative regulator of SOS downstream of ERK. Lack of novelty aside, I think it is important to show whether such feedbacks are occurring in this particular system.
It took quite a bit of thinking to get my head around what you might expect, and once I did - I think the results don't quite meet my theoretical predictions. The experiments are well done but it is not an obvious effect of RSK2 KD -, and the statistics seems very borderline to me (Figure 6H).
I also don't understand why the RSK2 KD doesn't increase ERK signalling (max increase or rate of activation) in the absence of MEK inhibition?
Can they firmly confirm the existence of this feedback loop? I am not so sure. Is it likely there? Probably? But it seems more experimental proof should be required here. Critically, there could be other explanation for this data.

We agree with the reviewer about the importance of clearly nailing down that RSK2 is indeed a negative regulator of ERK dynamics in our system. The modified version of the text and the figures now give a more detailed overview of the *RSK2* KD phenotype in the optoFGFR and in the optoSOS systems, which better highlights the evidence that RSK2 mediates a NFB.

1. Feature-based extraction of ERK dynamics shows that *RSK2* KD leads to the strongest prolongation of the response duration (ERKpostStim, Figure 4D, EV3A,B), which suggests a role in ERK adaptation, consistently with the role of RSK2 in a NFB. This data was shown in supplementary figure before but is now in the main figure.

2. *RSK2* KD, as well as *CRAF* and *ERK2* KD, led to a significant reduction of oscillatory ERK dynamics in the optoFGFR system (Figure 4F), which suggests a role of the RSK2-mediated NFB and the ERK-RAF NFB in the formation of these oscillations.
3. OptoSOS-induced ERK dynamics do not display oscillatory responses (Figure 5D, EV4B). In addition, *RSK2* KD in optoSOS-induced cells does not display slower adaptation (Figure 5F, EV4F,G). This new observation was possible thanks to the addition of new replicates of the siRNA screen in the optoSOS system, to which we added the *RSK2* KD.

About the drug responses, we now added experiments with additional MAPK inhibitors (Figure 6) which recapitulate the same results obtained with the MEK inhibitor shown in the previous version of the paper. This strengthens the concept that RSK2 perturbation can increase the efficiency of MAPK network inhibitors, allowing to homogeneously reduce ERK activity.

As to the question why RSK2 perturbation does not increase ERK rate of activation or amplitude, we do not have a clear mechanistic insight here. One important clue is that *RSK2* KD leads to lower ERK activity amplitude in the optoSOS system as well, suggesting that RSK2 also regulates the network below RAS (Figure 5F,G, EV4F). The interpretation of our results is based on the previously published finding that RSK2 acts as a NFB at the level of SOS (Saha et al. 2012; Douville and Downward 1997). However, we cannot formally exclude another modality of RSK2-mediated MAPK network regulation that might involve interactions that have not yet been documented. We now state this more clearly in the text.

That said, I am not at all convinced by the differential analysis of RSK2KD in optoFGFR vs optoSOS. While certainly the data suggests RSK2 could be involved in negative feedback, I do not see how they decided to put it at the level of SOS based on these experiments. Could it not just be that the signaling downstream of FGFR vs SOS is different in magnitude and/or type (number of effectors which interact with ERK signalling up- and down-stream?)
I think this is a major issue. Despite the fact it probably does exist, I don't think the authors have actually proven it here.

As discussed above, we have put the RSK2 negative feedback at the level of SOS because this has been previously documented in different cell systems (Douville and Downward 1997; Saha et al. 2012).

About the comparison of drug perturbations in optoFGFR versus optoSOS systems (Figure 6A-D), or optoFGFR in absence/presence of RSK2 perturbation (Figure 6E-H, EV5C-F), we agree with the reviewer that the effects are more evident in the optoFGFR versus optoSOS experiment. This might suggest that other regulation layers in addition to the RSK2 feedback are important to produce consistent ERK dynamics under node perturbation. This is now discussed in the text.

About the potential difference in magnitude/type of signaling downstream of optoFGFR and optoSOS, optoFGFR indeed activates PI3K/AKT and Ca^{2+} signaling which can feedback on ERK through crosstalks. However, we have shown that using minimal light inputs to get maximal ERK amplitude, we can evoke similar ERK amplitude using both optogenetic systems (Figure

5E). We assume that ERK dynamics evoked by both optogenetic systems identically control ERK-dependent downstream processes.

Our data interpretation is based on currently published data, but we cannot formally exclude other scenario that might involve unknown interactions in the MAPK network. We clearly state this in our manuscript.

Reviewer #2:

This manuscript describes a study on single cell ERK dynamics, specifically how a host of proteins that when knocked down influence the dynamics as controlled by an optogenetic FGFR system. The main impactful claim of the work is that ERK dynamics are robust to inhibition, unless a negative feedback loop they found involving RSK (already known) is targeted along with direct inhibition of the ERK pathway (MEK inhibition). This idea that the negative feedback loops lead to robustness of the ERK pathway is not new (as cited by the authors), although to my knowledge nobody has shown specifically that targeting the RSK negative feedback loop is key.
That being said, I found the evidence for this claim to be weak. First, the inhibitor doses being used were very high, so the possibility that off-target effects of the RSK and MEK inhibitors are leading to the varying robustness observations are high. Moreover, the MEK inhibitor being used, U0126, is an older one that has less specificity than newer generation ones.

We understand the reviewer's concerns about possible off target effect of the drugs at high concentrations. However, the concentrations used were selected based on previously published work and constrained by experimental observation to the concentrations required for strong ERK response inhibition.
For the experiments in optogenetic systems (in fibroblasts) with the RSK inhibitor SL0101, we used 100 µM as it was reported to be the working concentrations mostly used to block RSK (Roffé et al. 2015). In an exploration phase, we conducted experiments with 50 µM SL0101, but did not see an effect of the drug on ERK dynamics in combination with the other inhibitors. Even though SL0101 is not specific to the RSK2 isoform, the SL0101 perturbation (Figure EV5C-F) led to an identical phenotype than *RSK2* KD (Figure 6E-H). We thus believe that the concentration of SL0101 used was appropriate. For the MEK inhibitor (U0126), we used the same concentrations range than the one used by Sturm et al. (Sturm et al. 2010) and observed that full ERK inhibition required 50 µM U0126, which did not affect cell viability during our short-term experiments. We were quite surprised to see that at rather high U0126 concentrations, a subset of cells was still able to response to the stimulation (Figure EV5A optoFGFR, EV5B optoFGFR + *CTRL* KD). However, we observed that 10 µM U0126 was able to suppress ERK amplitudes in the optoSOS system (Figure 6B-D, Figure EV5A optoSOS) or in the optoFGFR system in combination with *RSK2* KD (Figure 6F-H, Figure EV5B optoFGFR + *RSK2* KD) or with 100 µM SL0101 (Figure EV5D-F).

We agree that U0126 is a quite old inhibitor which has been replaced by a newer generation of MEK inhibitors. We selected this inhibitor as it was previously used by Sturm et al. to evaluate the role of the ERK-RAF NFB in buffering against MEK perturbations. We now provide data for two additional MAPK inhibitors: the B/CRAF inhibitor (RAF709) and the ERK inhibitor (SCH772984). These are new generation inhibitors which allow us to probe the RAF and ERK nodes in addition to MEK. As for the MEK inhibitor, we observed that bypassing the RSK2-mediated NFB with optoSOS inputs, or perturbation of RSK2, led to an increase in efficiency of the B/CRAF and the ERK inhibitors. These results strengthen our observations that the RSK2-mediated NFB plays a role in the production of consistent ERK dynamics despite node perturbation. Results have been added to Figure 6 and Figure EV5.

For the MCF10A experiments, 100 µM SL0101 was indeed toxic to cells. We thus lowered the concentrations of SL0101 to 50 µM.

In Fig. 6, the choice of time point at which ERK activity effects of the inhibitor is evaluated may have a significant impact on the claim of decreased robustness. RSK knockdown only decreases the IC50 for the MEK inhibitor for ERK activity at this fixed time point by two-fold---what is the uncertainty in that estimate?

We agree that depending on the selected time points, the ERK responses will have different amplitudes, which might affect the difference in EC_{50} between the optoFGFR versus optoSOS system (Figure 6C) or between the optoFGFR system with *CTRL* KD versus *RSK2* KD (Figure 6G). To investigate this, we repeated the analysis of the optoFGFR versus optoSOS systems selecting different time points. We observed slight differences in the EC_{50} values but not change in the general trend (see Figure R1 added for the reviewer's convenience). ERK responses in the optoSOS system resulted in a lower EC_{50} for all timepoints and for the three MAPK inhibitors.

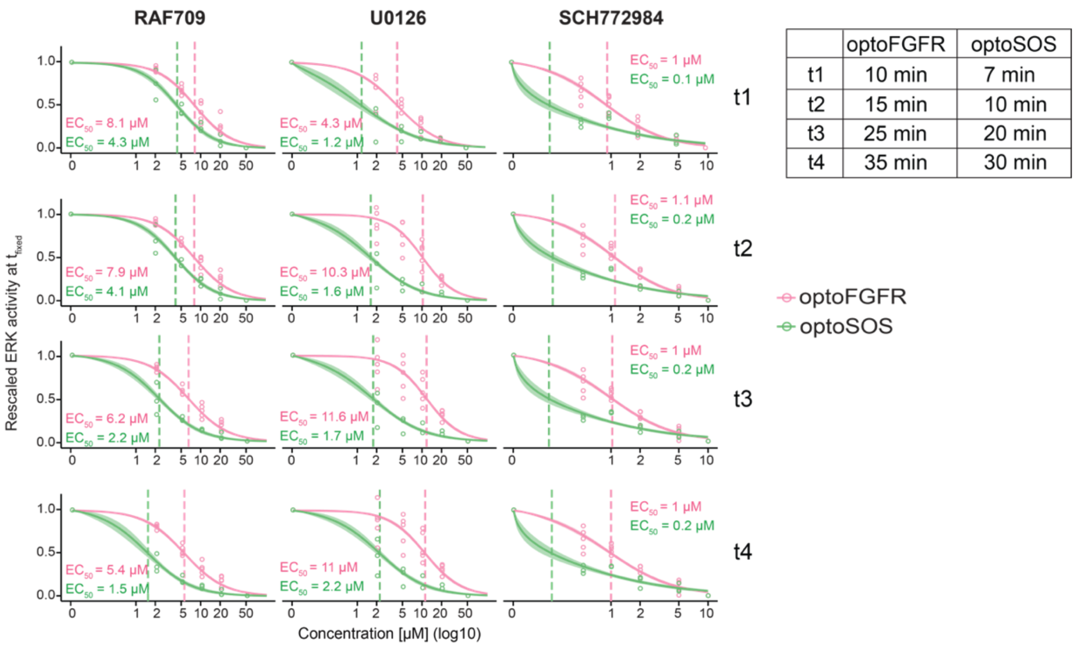

	optoFGFR	optoSOS
t1	10 min	7 min
t2	15 min	10 min
t3	25 min	20 min
t4	35 min	30 min

Figure R1: Comparison of MAPK inhibitors EC_{50} for different timepoints. ERK responses under increasing concentrations of MAPK inhibitors were extracted at different timepoints (t1, t2, t3, t4) from single-cell trajectories under sustained high optoFGFR (D = 18 mJ/cm^2) or optoSOS (D = 0.6 J/cm^2) inputs. A Hill function was fit to the normalized mean ERK activity (N_{min} = 200 cells per condition from three technical replicates). Shaded area indicates the 95% CI and dashed lines the EC_{50}.

And how does the RSK knockdown (or inhibition) affect cell viability, in more standard drug response assays? These are significant concerns that cast doubt upon the main impactful claim of the paper.

As the measurement of faithful ERK-KTR responses requires cells with healthy morphology, we always examined several fields of view to make sure that we work with healthy cells exhibiting a characteristic morphology throughout the experiment. Figure R2 displays some FOVs from the experiments presented in Figure 6, showing a comparison of cells treated

with *CTRL* KD versus *RSK2* KD (Figure R2A) or cells with or without RSK inhibition (Figure R2B). These images display healthy cells at the beginning and throughout the experiment. In addition, the morphology of the cells did not differ between optoFGFR with *CTRL* or *RSK2* KD, or between optoFGFR without or with 100 µM SL0101.

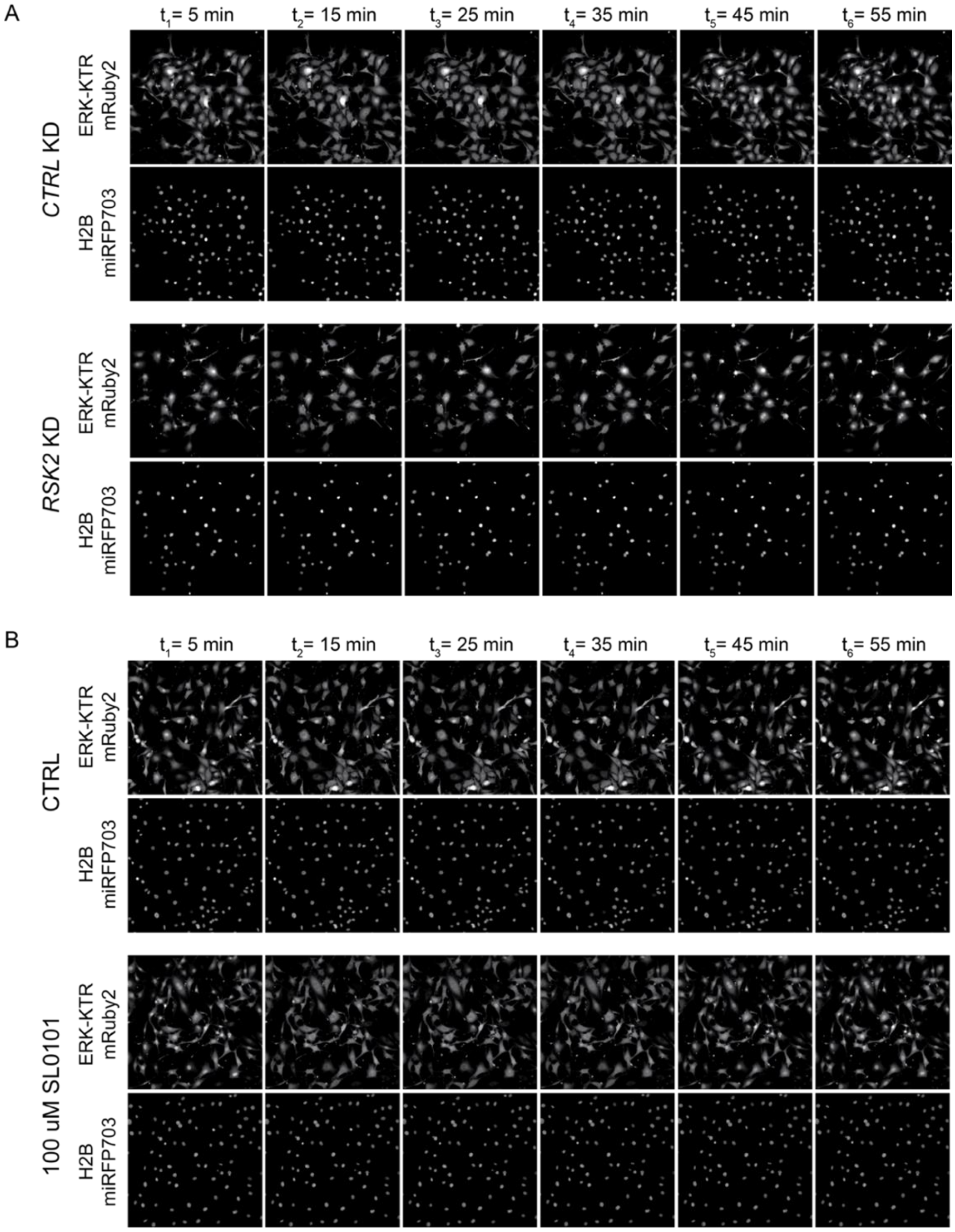

Figure R2: ERK-KTR and H2B time-lapse images under RSK2 perturbation. (A) NIH3T3 cells imaged at 72h hours post transfection with the non-targeting siRNA (*CTRL*) or a siRNA targeting *RSK2*. Cells were reversed transfected at a density of 3 x 10^2 cells per well in a 96-w plate and incubated for 72h at 37°C with 5% CO_2. ERK-KTR and H2B were imaged with a 20x air objective. Cells were stimulated at 2 minutes interval between t = 5 minutes and t = 33 minutes (D = 18 mJ/cm^2). **(B)** NIH3T3 cells were seeded at a density of 1.5 x 10^3 cells per well and incubated for 24h at 37°C with 5% CO_2. Cells were starved for 4h in absence or presence of 100 µM SL0101. ERK-KTR and H2B were imaged with a 20x air objective. Cells were stimulated at 2 min interval between t = 5 minute and t = 33 minutes (D = 18 mJ/cm^2).

We also evaluated cell viability under RSK2 perturbation by quantifying the number of cells post perturbation. In the case of the *RSK2* KD in NIH3T3 cells, we counted the number of cells in the well at 72h post seeding (3 x 10^2 cells per well) at the same time than transfection. We observed five time more cells in the *RSK2* KD well, suggesting that *RSK2* KD does not result in increased cell death within the experiment duration. However, consistently with the previously documented role of RSK2 on cell cycle regulation (Yoo et al. 2015), we observed more cells in the *CTRL* KD than in *RSK2* KD (Figure R3A). For SL0101, we conducted a time-course cell viability assay in MCF10A overexpressing ErbB2. We treated the cells with 50 µM SL0101 and evaluated the number of cells at different timepoints post addition of the drug (Figure R3B). We observed that, consistently with the role of RSK2 in cell proliferation (Yoo et al. 2015), the number of cells only increasing to a limited extend when treated with SL0101 compared to the control (DMSO). However, SL0101 treatment did not result in a reduction of cell number over the observed period, suggesting that the concentration used does not induce increased cell death.

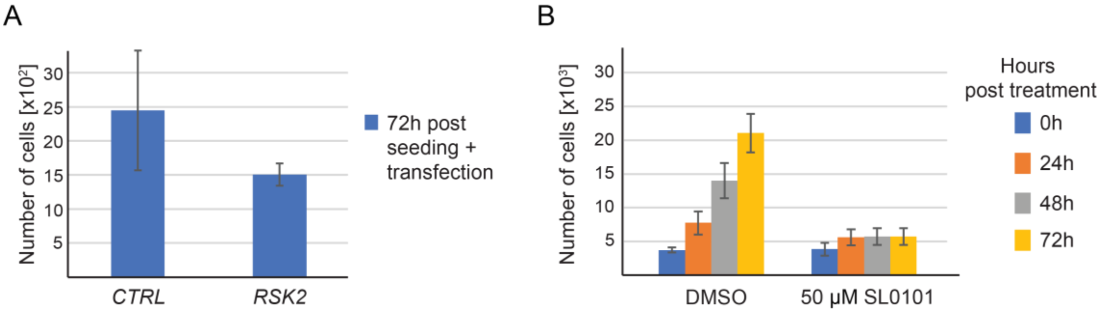

Figure R3: Quantification of cell viability under RSK2 perturbation. (A) Number of NIH3T3 cells at 72h hours post transfection with the non-targeting siRNA (*CTRL*) or a siRNA targeting *RSK2*. Cells were reversed transfected at a density of 3 x 10^2 cells per well in a 96-w plate and incubated for 72h at 37°C with 5% CO_2. After 72h, images of the nuclear marker (H2B*miRFP*) were then taken with a 10x objective. Number of cells per condition was quantified from 2 technical replicates. **(B)** Number of MCF10A overexpressing ErbB2 cells at different timepoints post treatment with DMSO or 50 µM SL0101. Cells were seeded at 3 x 10^3 cells per well in a 96-w plate and incubated in growth medium for 48h at 37°C with 5% CO_2. Medium was then replaced by starving medium before addition of the drugs. Cells were further incubated at 37°C with 5% CO_2 and images of the nuclear channel (H2B*miRFP*) were taken with a 20x objective every 24h.

That being said, the expansive knockdown data set on how various protein levels influence ERK dynamics I thought was very useful and informative and would be a welcome addition to the literature on the topic. The method for selecting which proteins to knockdown was interesting and useful.

We thank the reviewer for his/her supporting comments.

Reviewer #3:

This manuscript from Dessauges and colleagues presents a deep analysis of ERK activity dynamics, using a combination of optogenetic tools, siRNA-based knockdowns, and various quantitative analyses. A major conclusion is that the dynamics of growth factor receptor-stimulated ERK activity are largely unperturbed by knockdown of a majority of the pathway components. A parallel analysis with a second optogenetic stimulation that operates downstream of the receptor is used to demonstrate that this robustness arises from feedback to the receptor level, rather than feedback operating within the Ras-Raf-MEK-ERK cascade. The authors then look further into the specific mechanism of feedback from ERK to receptor via RSK2, and attempt to make the case that targeting this negative feedback route can reduce the robustness of the ERK dynamics and increase the efficacy of MEK inhibition as a pathway-targeting strategy.
The first five figures of this work are extremely impressive - a technical tour de force that sets a new standard for the entire field. Compared to the western blot-based studies that still dominate the signaling literature, this paper demonstrates nothing short of a quantum leap in the ability to collect carefully validated, highly quantitative data on a large scale, producing a dataset with great power to better understand growth factor signaling at the systems level. Every step in the process is carefully validated and clearly presented. The large dataset reveals the single-cell intricacies of MAPK signaling in much greater depth than any other work and will undoubtedly power many modeling studies to come. We are extremely enthusiastic to see this data and analysis published quickly.

We are grateful for the supporting comments of the reviewer.

We had more reservations about the last two figures of the paper, and the conclusions drawn concerning the effect of RSK2-mediated feedback on the robustness of the pathway. As detailed further in the points below, the concept of robustness as it applies in this case is not well defined, and a number of the individual experiments don't provide strong support for the conclusions that are being made. We found this section much harder to get through without stumbling on various inconsistencies, and we think it needs some revision to better justify the overall conclusions.

Major points

1. The concept of "robustness" as it applies to ERK outputs is not clearly defined, especially in the section from 388-409. It is difficult to tell whether robustness is being equated with the decrease in heterogeneous ERK behavior, or a lower IC50 for MEK inhibition. There are important differences between these characteristics, and we were not sure whether the focus is meant to be on biological robustness (where diversity of cell phenotypes may be advantageous) or on pharmacological robustness (sensitivity to inhibition). Some clarification is needed here. Also, might it be a better representation of robustness, from the standpoint of pathway output, to look at the shallower dose response relationship seen in optoSOS rather than a simple change in IC50?

We thank the reviewer for highlighting our lack of clarity concerning the concept of robustness and giving us the opportunity to clarify what we meant. With this term , we

aimed to describe the ability of the MAPK network to produce consistent ERK dynamics (in terms of amplitude and shape) in presence of signaling node perturbations. We now have added a clear definition of what we mean with signaling robustness in the introduction. We also have rewritten the results part about Figures 6 and 7 to clarify that we are looking at the efficiency of the drugs to homogeneously reduce ERK amplitudes.

Regarding the evaluation of this MAPK signaling robustness, we were interested in evaluating how the RSK2-mediated NFB affect the efficiency of the MAPK inhibitors on suppressing ERK responses in all the cells of the population. We thus believe that looking at the effects of the inhibitors on ERK response amplitude distribution (Figure 6B,F, EV5D), the EC_{50} (Figure 6C,G, EV5E) and single-cell heterogeneity (Figure 6D,H, EV6F) provide an adequate evaluation. Observations made with these three metrics led to the hypothesis that targeting the RSK2-mediated feedback increases the efficiency of the pharmacological perturbation of B/CRAF, MEK or ERK. Please note that the B/CRAF inhibitor and the ERK inhibitor data were newly added. We believe that these additional analyses strengthen our hypothesis about the role of the RSK2-mediated feedback in MAPK signaling robustness.

2. The differences between optoSOS and optoFGFR in MEK inhibition are clear (Fig. 6C-D), but the effects of RSK2 knockdown (Fig. 6G-H), and inhibition (EV5C-D) are substantially weaker. There are other known negative feedbacks affecting receptor internalization (e.g. Sprouty, etc.) which likely account for the difference, but the narrative seems to focus on RSK2 over these other possibilities in a way that doesn't seem fitting with the relatively modest experimental results.

We agree with the reviewer's comment. This point should have been discussed more carefully. We now clearly state that the increase in efficiency of the different drugs is stronger in the optoSOS system versus optoFGFR than in the optoFGFR system + RSK2 perturbation versus CTRL, suggesting that RSK2 is important but not the only player involved in MAPK signaling robustness. We now discuss possible other explanation for the signaling robustness we observed in the discussion.

3. The effects of RSK2 feedback perturbation could be better understood if they were more connected to the modeling. The fact that inhibiting RSK2-based negative feedback both decreases amplitude and leads to higher sensitivity to MEK inhibition is somewhat counterintuitive. We assume this effect arises because of differences in "thresholds" of MEK/ERK activity needed to activate the two different feedbacks? It seems that the models could be used more here to gain some intuition into the observed RSK2 inhibition results. Alternatively, the authors' understanding of what is happening to decrease ERK amplitude/increase MEK inhibition sensitivity in RSK2-knockdown/inhibited cells could be more clearly explained.

We agree that being able to produce a model encompassing two negative feedbacks (ERK-RAF and ERK-RSK2-SOS) could have enabled a better understanding of the different phenotypes we observed. However, we could not fit a more complex model consisting of two negative feedbacks due to the lack of ability to identify a meaningful parameter space. Therefore, we tried to get a better understanding of the *RSK2* KD phenotype based on our experimental data (Figure 4D-F, EV3A,B, 5F,G, EV4F,G). Please note that we now described

more clearly the *RSK2* KD phenotype in response to optoFGFR and optoSOS inputs (see answer to reviewer 1) which should make our claims easier to understand.

Concerning the observation that *RSK2* KD alone induces a reduction in ERK amplitude in the optoFGFR system (Figure 4D, EV3A), we can only provide a data-driven hypothesis. We added the *RSK2* KD to the optoSOS perturbation screen. We observed that *RSK2* KD also induced a reduction of ERK amplitude in the optoSOS system (Figure 5F, EV4F), while it did not result in a prolonged ERK response (Figure 5F, EV4F,G) as observed in the optoFGFR system (Figure 4D, EV3A,B). Based on these observations, we concluded that RSK2, in addition to its role in the ERK-RSK2-SOS NFB, might regulate nodes downstream of RAS.

4. The section from line 440 to 460 relies heavily on interpreting the dispersion of cells within tSNE plots as "compact" or spread. It is now widely appreciated that tSNE plots can wildly distort distances between points, and this ambiguity makes it unclear how useful the dispersion of points in these visualizations can be in representing the heterogeneity of cells.

We thank the reviewer for pointing out this issue. We now reanalyzed our data to remove human bias in its interpretation. We have now clustered the CNN features to classify ERK trajectories in response to different perturbations, rather than identifying specific ERK trajectory clusters by visual examination of the tSNE plots (Figure 7E,I). This enabled to define specific clusters of ERK dynamics without human bias and to evaluate the heterogeneity of single-cell ERK trajectories in response to different perturbations (Figure 7F,J). Further, this enables to display the medoid and the 4 closest neighbor ERK trajectories for each cluster (Figure 7G,K). This approach captured similar trends in the data as presented before. It also allowed us to provide a more intuitive description of the single-cell heterogeneity of ERK dynamics under the different perturbations (Figure 7F,J).

5. The cell cycle experiment in Figure 7 is difficult to interpret. Since RSK has ERK-independent effect, it is difficult to know how much of combined treatment's effect can be attributed to reduced ERK dynamics rather than combined effects on cell cycle mediators downstream of ERK. While I understand the objective to demonstrate an effect at the cellular phenotype level, I don't think this result clearly adds to the intended point about targeting feedback to sensitize the ERK pathway.

We do agree with the reviewer that the role of RSK regulating cell-cycle mediators downstream of ERK makes it difficult to investigate the ERK-dependent role of the RSK2-mediated feedback perturbation on cell cycle entry. Even though we could observe a stronger suppression of Geminin activity in cells treated with 50 µM SL0101 + 1 µM U0126 than with 50 µM SL0101, the difference in effects of the single drug and combined drugs was indeed difficult to interpret. We thus propose to leave out this panel from this work.

6. The overall dataset makes it clear that the ERK network is quite resistant to perturbations of single genes. However, the strong effect of an ERK1+ERK2 knockdown also hints that the combined perturbations of the same node can cause quite large changes. This raises the question of how much of the apparent robustness is really due to feedback regulation, rather than simply to gene redundancy. While it seems most reasonable to save an in depth experimental analysis of combined knockdowns for a later study, this point does warrant

some discussion. Furthermore, there remains the possibility that some dosage compensation of expression levels could occur; although this seems unlikely given the timescale of the knockdowns, it deserves some mention as a caveat.

We agree with the reviewer that in our previous version of the manuscript we attributed too much weight to the role of the RSK2-mediated feedback in MAPK signaling robustness. We now state more carefully the fact that the RSK2-mediated feedback is an important factor, but not the only player. We also discuss the fact that the presence of additional feedbacks, the high isoforms redundancy and possible transcriptional compensation mechanisms might be involved.

Minor points

On line 429, is "potentiate" intended rather than "potential"?

Corrected

Lines 355-366 seem to have a redundant "light pulses".

Removed

Line 399 - U0126 "misspelled".

Corrected

The color scheme in figure 7J and K is confusing and seems like it may be mislabeled. The ordering of colors in the stacked bars doesn't match their numerical order (why is 4 on top of 5?). The similarities of colors used in the heatmap and those used to represent the different clusters is also a bit disorienting.

Following the above comments about the ERK-independent role of RSK on cell cycle regulation, we propose to leave out this panel from this work. However, we agree that the colors of the heatmap and cluster labels could have been better chosen.

Not much mention is made of the effects of knockdowns on basal state, other than DUSP6. It would be interesting and helpful to include a supplemental plot similar to Fig. 4D comparing changes in basal ERK activity.

We thank the reviewer for pointing out that an overview of the effects of the perturbations on basal ERK activity could be informative. We have now added an overview of the effects of the different perturbation on ERK baseline and ERK adaptation to the previously shown analysis of ERK amplitude, both in the optoFGFR system (Figure 4D, EV3A) and in the optoSOS system (Figure 5F, EV4F).

References

Blum, Y., Mikelson, J., Dobrzyński, M., et al. 2019. Temporal perturbation of ERK dynamics reveals network architecture of FGF2/MAPK signaling. *Molecular Systems Biology* 15(11), p. e8947.

Blüthgen, N. and Legewie, S. 2013. Robustness of signal transduction pathways. *Cellular and Molecular Life Sciences* 70(13), pp. 2259–2269.

Douville, E. and Downward, J. 1997. EGF induced SOS phosphorylation in PC12 cells involves P90 RSK-2. *Oncogene* 15(4), pp. 373–383.

Fritsche-Guenther, R., Witzel, F., Sieber, A., et al. 2011. Strong negative feedback from Erk to Raf confers robustness to MAPK signalling. *Molecular Systems Biology* 7, p. 489.

Gerosa, L., Chidley, C., Fröhlich, F., et al. 2020. Receptor-Driven ERK Pulses Reconfigure MAPK Signaling and Enable Persistence of Drug-Adapted BRAF-Mutant Melanoma Cells. *Cell Systems* 11(5), p. 478–494.e9.

Kanodia, J., Chai, D., Vollmer, J., et al. 2014. Deciphering the mechanism behind Fibroblast Growth Factor (FGF) induced biphasic signal-response profiles. *Cell Communication and Signaling* 12, p. 34.

Kholodenko, B.N., Hancock, J.F. and Kolch, W. 2010. Signalling ballet in space and time. *Nature Reviews. Molecular Cell Biology* 11(6), pp. 414–426.

Kiyatkin, A., van Alderwerelt van Rosenburgh, I.K., Klein, D.E. and Lemmon, M.A. 2020. Kinetics of receptor tyrosine kinase activation define ERK signaling dynamics. *Science Signaling* 13(645).

Roffé, M., Lupinacci, F.C., Soares, L.C., Hajj, G.N. and Martins, V.R. 2015. Two widely used RSK inhibitors, BI-D1870 and SL0101, alter mTORC1 signaling in a RSK-independent manner. *Cellular Signalling* 27(8), pp. 1630–1642.

Saha, M., Carriere, A., Cheerathodi, M., et al. 2012. RSK phosphorylates SOS1 creating 14-3-3-docking sites and negatively regulating MAPK activation. *The Biochemical Journal* 447(1), pp. 159–166.

Santos, S.D.M., Verveer, P.J. and Bastiaens, P.I.H. 2007. Growth factor-induced MAPK network topology shapes Erk response determining PC-12 cell fate. *Nature Cell Biology* 9(3), pp. 324–330.

Sturm, O.E., Orton, R., Grindlay, J., et al. 2010. The mammalian MAPK/ERK pathway exhibits properties of a negative feedback amplifier. *Science Signaling* 3(153), p. ra90.

Yoo, S.-M., Cho, S.J. and Cho, Y.-Y. 2015. Molecular targeting of erks/rsk2 signaling axis in cancer prevention. *Journal of cancer prevention* 20(3), pp. 165–171.

1st Revision - Editorial Decision — 6th May 2022

Thank you for sending us your revised manuscript. We have now heard back from two of the three reviewers who agreed to evaluate your study. Unfortunately, after a series of reminders, we did not obtain a report from Reviewer #1. In the interest of time, I prefer to make a decision now rather than further delay the process. As you will see, the reviewers are satisfied with the modifications made and think that the study is now suitable for publication.

Before we can formally accept your manuscript, we would ask you to address the following issues:
1. The remaining minor concerns of Reviewer #2.

On a more editorial level:

REFEREE REPORTS

Reviewer #2:

The authors have solidly addressed my concerns raised during review (and it would seem of the other reviewers as well). The addition of multiple inhibitors and in general a more robust dataset will help give the scientific community confidence in the interesting claims of this paper. The dataset itself will likely be mined by many others for modeling and new biological exploration.

Only one minor point from the introduction: Ras GTPase activating proteins (GAPs) do not activate Ras signaling (it is confusing I know), but the text reads as so. GAPs inactivate Ras.

Reviewer #3:

The authors have dealt quite thoroughly with the reviewers' comments. While it is still not exactly clear how the RSK negative feedback loop exerts the observed effects, I think that is outside of the scope for this paper, and the effect is now well documented enough that it will be of use to researchers following up on these findings. On the whole I think this paper is extremely exciting and will be of enormous value to the field. I look forward to seeing it published.

The authors have made all requested editorial changes.

Accepted

17th May 2022

Thank you again for sending us your revised manuscript. We are now satisfied with the modifications made and I am pleased to inform you that your paper has been accepted for publication.

EMBO Press Author Checklist

Corresponding Author Name: Olivier Pertz	
Journal Submitted to: Molecular Systems Biology	
Manuscript Number: MSB-2021-10670	

USEFUL LINKS FOR COMPLETING THIS FORM
The EMBO Journal - Author Guidelines
EMBO Reports - Author Guidelines
Molecular Systems Biology - Author Guidelines
EMBO Molecular Medicine - Author Guidelines

Reporting Checklist for Life Science Articles (updated January 2022)
This checklist is adapted from Materials Design Analysis Reporting (MDAR) Checklist for Authors. MDAR establishes a minimum set of requirements in transparent reporting in the life sciences (see Statement of Task: 10.31222/osf.io/9sm4x). Please follow the journal's guidelines in preparing your manuscript.
Please note that a copy of this checklist will be published alongside your article.

Abridged guidelines for figures
1. Data
The data shown in figures should satisfy the following conditions:
- the data were obtained and processed according to the field's best practice and are presented to reflect the results of the experiments in an accurate and unbiased manner.
- ideally, figure panels should include only measurements that are directly comparable to each other and obtained with the same assay.
- plots include clearly labeled error bars for independent experiments and sample sizes. Unless justified, error bars should not be shown for technical replicates.
- if n<5, the individual data points from each experiment should be plotted. Any statistical test employed should be justified.
- Source Data should be included to report the data underlying figures according to the guidelines set out in the authorship guidelines on Data Presentation.

2. Captions
Each figure caption should contain the following information, for each panel where they are relevant:
- a specification of the experimental system investigated (eg cell line, species name).
- the assay(s) and method(s) used to carry out the reported observations and measurements.
- an explicit mention of the biological and chemical entity(ies) that are being measured.
- an explicit mention of the biological and chemical entity(ies) that are altered/varied/perturbed in a controlled manner.
- the exact sample size (n) for each experimental group/condition, given as a number, not a range;
- a description of the sample collection allowing the reader to understand whether the samples represent technical or biological replicates (including how many animals, litters, cultures, etc.).
- a statement of how many times the experiment shown was independently replicated in the laboratory.
- definitions of statistical methods and measures:
  - common tests, such as t-test (please specify whether paired vs. unpaired), simple χ2 tests, Wilcoxon and Mann-Whitney tests, can be unambiguously identified by name only, but more complex techniques should be described in the methods section;
  - are tests one-sided or two-sided?
  - are there adjustments for multiple comparisons?
  - exact statistical test results, e.g., P values = x but not P values < x;
  - definition of 'center values' as median or average;
  - definition of error bars as s.d. or s.e.m.

Please complete ALL of the questions below.
Select "Not Applicable" only when the requested information is not relevant for your study.

Materials

Newly Created Materials	Information included in the manuscript?	In which section is the information available? (Reagents and Tools Table, Materials and Methods, Figures, Data Availability Section)
New materials and reagents need to be available; do any restrictions apply?	Yes	The plasmids used to generate the optogenetic actuators/ERK biosensors circuits used in this study are described in the Materials and Methods

Antibodies	Information included in the manuscript?	In which section is the information available? (Reagents and Tools Table, Materials and Methods, Figures, Data Availability Section)
For **antibodies** provide the following information:<br>- Commercial antibodies: RRID (if possible) or supplier name, catalogue number and or/clone number<br>- Non-commercial: RRID or citation	Yes	Information given in Materials and Methods

DNA and RNA sequences	Information included in the manuscript?	In which section is the information available? (Reagents and Tools Table, Materials and Methods, Figures, Data Availability Section)
**Short novel DNA or RNA including primers, probes**: provide the sequences.	Yes	siRNA sequences are available in Data Availability Section

Cell materials	Information included in the manuscript?	In which section is the information available? (Reagents and Tools Table, Materials and Methods, Figures, Data Availability Section)
**Cell lines:** Provide species information, strain. Provide accession number in repository **OR** supplier name, catalog number, clone number, and/**OR** RRID.	Yes	Names of cell lines are in Materials and Methods
**Primary cultures:** Provide species, strain, sex of origin, genetic modification status.	Not Applicable	
Report if the cell lines were recently **authenticated** (e.g., by STR profiling) and tested for mycoplasma contamination.	Not Applicable	

Experimental animals	Information included in the manuscript?	In which section is the information available? (Reagents and Tools Table, Materials and Methods, Figures, Data Availability Section)
**Laboratory animals or Model organisms:** Provide species, strain, sex, age, genetic modification status. Provide accession number in repository **OR** supplier name, catalog number, clone number, **OR** RRID.	Not Applicable	
**Animal observed in or captured from the field:** Provide species, sex, and age where possible.	Not Applicable	
Please detail **housing and husbandry conditions**.	Not Applicable	

Plants and microbes	Information included in the manuscript?	In which section is the information available? (Reagents and Tools Table, Materials and Methods, Figures, Data Availability Section)
**Plants:** provide species and strain, ecotype and cultivar where relevant, unique accession number if available, and source (including location for collected wild specimens).	Not Applicable	
**Microbes:** provide species and strain, unique accession number if available, and source.	Not Applicable	

Human research participants	Information included in the manuscript?	In which section is the information available? (Reagents and Tools Table, Materials and Methods, Figures, Data Availability Section)
If collected and within the bounds of privacy constraints report on age, sex and gender or ethnicity for all study participants.	Not Applicable	

Core facilities	Information included in the manuscript?	In which section is the information available? (Reagents and Tools Table, Materials and Methods, Figures, Data Availability Section)
If your work benefited from core facilities, was their service mentioned in the acknowledgments section?	Yes	We thanked the Microscopy Imaging Center from the Univeristy of Bern in the Acknowledgements

Design

Study protocol	Information included in the manuscript?	In which section is the information available? (Reagents and Tools Table, Materials and Methods, Figures, Data Availability Section)
If study protocol has been **pre-registered, provide DOI in the manuscript**. For clinical trials, provide the trial registration number **OR** cite DOI.	Not Applicable	
Report the **clinical trial registration number** (at ClinicalTrials.gov or equivalent), where applicable.	Not Applicable	

Laboratory protocol	Information included in the manuscript?	In which section is the information available? (Reagents and Tools Table, Materials and Methods, Figures, Data Availability Section)
Provide DOI OR other citation details if **external detailed step-by-step protocols** are available.	Yes	References to previously published protocoles are given in Materials and Methods

Experimental study design and statistics	Information included in the manuscript?	In which section is the information available? (Reagents and Tools Table, Materials and Methods, Figures, Data Availability Section)
Include a statement about **sample size** estimate even if no statistical methods were used.	Yes	Sample size is given in the figure legends
Were any steps taken to minimize the effects of subjective bias when allocating animals/samples to treatment (e.g. **randomization procedure**)? If yes, have they been described?	Not Applicable	
Include a statement about **blinding** even if no blinding was done.	Not Applicable	
Describe **inclusion/exclusion criteria** if samples or animals were excluded from the analysis. Were the criteria pre-established?<br><br>If sample or data points were omitted from analysis, report if this was due to attrition or intentional exclusion and provide justification.	Yes	Criteria used to filter data are described in Materials and Methods
For every figure, are **statistical tests** justified as appropriate? Do the data meet the assumptions of the tests (e.g., normal distribution)? Describe any methods used to assess it. Is there an estimate of variation within each group of data? Is the variance similar between the groups that are being statistically compared?	Yes	Staticial tests are described in the figure legends and Materials and Methods

Sample definition and in-laboratory replication	Information included in the manuscript?	In which section is the information available? (Reagents and Tools Table, Materials and Methods, Figures, Data Availability Section)
In the figure legends: state number of times the experiment was **replicated** in laboratory.	Yes	Information about the number of replicates is given in the figure legends
In the figure legends: define whether data describe **technical or biological replicates**.	Yes	Information about the kind of replicates is given in the figure legends

Ethics

Ethics	Information included in the manuscript?	In which section is the information available? (Reagents and Tools Table, Materials and Methods, Figures, Data Availability Section)
Studies involving **human participants**: State details of **authority granting ethics approval** (IRB or equivalent committee(s), provide reference number for approval.	Not Applicable	
Studies involving **human participants**: Include a statement confirming that **informed consent** was obtained from all subjects and that the experiments conformed to the principles set out in the WMA Declaration of Helsinki and the Department of Health and Human Services Belmont Report.	Not Applicable	
Studies involving **human participants:** For publication of **patient photos**, include a statement confirming that consent to publish was obtained.	Not Applicable	
Studies involving experimental **animals**: State details of **authority granting ethics approva**l (IRB or equivalent committee(s), provide reference number for approval. Include a statement of compliance with ethical regulations.	Not Applicable	
Studies involving **specimen and field samples:** State if relevant **permits** obtained, provide details of authority approving study; if none were required, explain why.	Not Applicable	

Dual Use Research of Concern (DURC)	Information included in the manuscript?	In which section is the information available? (Reagents and Tools Table, Materials and Methods, Figures, Data Availability Section)
Could your study fall under dual use research restrictions? Please check biosecurity documents and list of **select agents and toxins** (CDC): https://www.selectagents.gov/sat/list.htm	Not Applicable	
If you used a select agent, is the security level of the lab appropriate and reported in the manuscript?	Not Applicable	
If a study is subject to dual use research of concern regulations, is the name of the **authority granting approval and reference number** for the regulatory approval provided in the manuscript?	Not Applicable	

Reporting

The MDAR framework recommends adoption of discipline-specific guidelines, established and endorsed through community initiatives. Journals have their own policy about requiring specific guidelines and recommendations to complement MDAR.

Adherence to community standards	Information included in the manuscript?	In which section is the information available? (Reagents and Tools Table, Materials and Methods, Figures, Data Availability Section)
State if relevant guidelines or checklists (e.g., **ICMJE, MIBBI, ARRIVE, PRISMA**) have been followed or provided.	Not Applicable	
For **tumor marker prognostic studies,** we recommend that you follow the **REMARK** reporting guidelines (see link list at top right). See author guidelines, under 'Reporting Guidelines'. Please confirm you have followed these guidelines.	Not Applicable	
For **phase II and III randomized controlled trials**, please refer to the **CONSORT** flow diagram (see link list at top right) and submit the CONSORT checklist (see link list at top right) with your submission. See author guidelines, under 'Reporting Guidelines'. Please confirm you have submitted this list.	Not Applicable	

Data Availability

Data availability	Information included in the manuscript?	In which section is the information available? (Reagents and Tools Table, Materials and Methods, Figures, Data Availability Section)
Have **primary datasets** been deposited according to the journal's guidelines (see 'Data Deposition' section) and the respective accession numbers provided in the Data Availability Section?	Yes	All data and codes used in this study have been placed on a Mendeley repository with a link given in the Data availability section
Were **human clinical and genomic datasets** deposited in a public access-controlled repository in accordance to ethical obligations to the patients and to the applicable consent agreement?	Not Applicable	
Are **computational models** that are central and integral to a study available without restrictions in a machine-readable form? Were the relevant accession numbers or links provided?	Yes	All material and codes used for the modeling part are available on github, link given in the Data availability section
If publicly available data were reused, provide the respective **data citations in the reference list**.	Not Applicable	
